# A non-catalytic N-terminus domain of WRN prevents mitotic telomere deprotection

**DOI:** 10.1038/s41598-023-27598-0

**Published:** 2023-01-12

**Authors:** Diana Romero-Zamora, Makoto T. Hayashi

**Affiliations:** 1 Graduate School of Biostudies, Kyoto University, Yoshida-Konoe, Sakyo, Kyoto, 606-8501 Japan; 2 IFOM-KU Joint Research Laboratory, Graduate School of Medicine, Kyoto University, Yoshida-Konoe, Sakyo, Kyoto, 606-8501 Japan; 3 IFOM ETS, The AIRC Institute of Molecular Oncology, Via Adamello 16, 20139 Milan, Italy

**Keywords:** Chromosomes, Telomeres, Chromosomes, Telomeres, DNA damage response

## Abstract

Telomeric ends form a loop structure (T-loop) necessary for the repression of ATM kinase activation throughout the normal cell cycle. However, cells undergoing a prolonged mitotic arrest are prone to lose the T-loop, resulting in Aurora B kinase-dependent mitotic telomere deprotection, which was proposed as an anti-tumor mechanism that eliminates precancerous cells from the population. The mechanism of mitotic telomere deprotection has not been elucidated. Here, we show that WRN, a RECQ helicase family member, can suppress mitotic telomere deprotection independently of its exonuclease and helicase activities. Truncation of WRN revealed that N-terminus amino acids 168–333, a region that contains a coiled-coil motif, is sufficient to suppress mitotic telomere deprotection without affecting both mitotic Aurora B-dependent spindle checkpoint and ATM kinase activity. The suppressive activity of the WRN^168–333^ fragment is diminished in cells partially depleted of TRF2, while WRN is required for complete suppression of mitotic telomere deprotection by TRF2 overexpression. Finally, we found that phosphomimetic but not alanine mutations of putative Aurora B target sites in the WRN^168–333^ fragment abolished its suppressive effect. Our findings reveal a non-enzymatic function of WRN, which may be regulated by phosphorylation in cells undergoing mitotic arrest. We propose that WRN enhances the protective function of TRF2 to counteract the hypothetical pathway that resolves the mitotic T-loop.

## Introduction

Telomeres are composed of repetitive DNA sequences and telomere-associated proteins. They cap the end of eukaryotic chromosomes and prevent activation of the DNA damage pathway (DDR), thus safeguarding the genome from instability^1^. In humans and among many model organisms, the protective role of telomeres relies on the ability of the telomeric ends to invade telomeric dsDNA resulting in a displacement loop (D-loop)^2,3^, which is stabilized mainly by TRF2, a member of the six telomere-specific proteins that comprise the shelterin complex^4^. As a result, the stabilized D-loop forms a telomere loop (T-loop), which masks the chromosome terminus from being recognized as a DNA double-strand break^1^. The T-loop is an essential structure encompassing the “closed-state” of the telomeres, preventing DDR activation through the cell cycle and inhibiting chromosome fusions which are one of the consequences of DDR. The absence of a T-loop at TRF2-positive telomeres results in an “intermediate-state,” in which telomeres are prone to ATM-dependent checkpoint activation but are still able to prevent telomere fusions^5–7^. This status is achieved by retaining sufficient TRF2 levels at exposed telomeric ends and, thus, prevents undesired DNA repair through the Non-Homologous End Joining (NHEJ) pathway^8,9^. The “intermediate-state” occurs in telomerase-negative cells undergoing replicative telomere shortening, triggering ATM kinase to phosphorylate diverse substrates located at the telomeres, including histone H2AX^10^. The resulting γ-H2AX serves as a marker of telomere dysfunction-induced foci (TIFs)^11^, which is also observed in non-fused long telomeres upon partial depletion of TRF2 by shRNA-mediated knockdown^6^. On the other hand, complete loss of TRF2 function results in an “uncapped-state”, in which telomeres are prone to end-to-end chromosome fusions leading to chromosomal instability as a consequence^6,12^. Hence, effective protection of chromosome ends requires sufficient functional TRF2 that thoroughly represses ATM and NHEJ activation through T-loop formation.

Furthermore, previous studies have shown that cells arrested in prometaphase/metaphase due to spindle assembly checkpoint (SAC) activation or anaphase-promoting complex (APC) inhibition experience an event known as mitotic telomere deprotection^8,13,14^. This event is characterized by mitotic arrest-dependent TIF (MAD-TIF) formation on mitotic chromosomes. Super-resolution microscopy techniques, such as STORM and Airyscan types, effectively visualized mitotically deprotected telomeres and demonstrated that T-loop unfolding induces ATM activation during mitotic arrest^8^. These studies also reported that T-loop unfolding and resulting MAD-TIFs depend on Aurora B kinase^8,13^, an essential component of the chromosome passenger complex (CPC)^15^. Consequently, mitotic cells harboring MAD-TIFs with increased DDR signaling are subjected to cell death during mitotic arrest or p53-dependent cell cycle arrest in the following cell cycle^13,14,16^. A spontaneous mitotic arrest occurs in p53-compromised human fibroblasts undergoing telomere crisis, which is characterized by end-to-end chromosome fusions and massive cell death; mitotic telomere deprotection was shown to underlie this cell death pathway during telomere crisis^14^. Similarly, mitotic telomere deprotection can be achieved by treating fibroblasts, epithelial cells, and cancer cells with drugs that induce mitotic arrest (e.g., dimethylenastron, vinblastine, taxol, and colcemid) ^6,13^. However, the factors underlying the mechanism of mitotic telomere deprotection have not been fully elucidated.

According to a growing body of research, the D-loop structure has been found to be a favorite DNA substrate for specific helicases such as RTEL1 and RecQ helicase family members, BLM and WRN^17–20^. The preference of RTEL1 to target D-loop substrates contributes to the resolution of the T-loop during the telomere replication process, which is needed for the successful synthesis of the telomeric DNA lagging strand and for preventing replication stress^21^. We, therefore, speculated that RecQ helicases could play a role in telomere regulation by targeting the T-loop in distinct cellular processes.

Abnormal mutations in the gene encoding WRN helicase result in the aging-associated WRN syndrome characterized by a rapid telomere shortening and impaired genomic maintenance^22,23^. WRN contains helicase and 3'-5' exonuclease activities that help telomere maintenance and in-vitro processing of telomeric D-loop substrates^24^. Interaction with TRF2 stimulates the ATPase activity of WRN to help telomeric recombination processes^18,20,25^, whereas interaction with TRF1 negatively regulates WRN exonuclease activity for the dissolution of D-loop substrates^19^. Overall, the interaction of WRN with telomeric proteins indicates its relevance for supporting genome stability through its involvement in telomere maintenance. The recognized roles of WRN during telomere replication and its constitutive expression throughout the cell cycle raise the possibility that WRN could play a role during mitotic telomere deprotection, which remains uncharacterized.

Here, we investigated WRN involvement in mitotic telomere deprotection. We observed that depletion and exogenous expression of WRN increase and decrease the MAD-TIF rate, respectively, suggesting that WRN functions as a suppressor of mitotic telomere deprotection. We found that both exonuclease- and helicase-dead WRN could suppress mitotic telomere deprotection, indicating that enzymatic activities of WRN are dispensable. Experiments using WRN truncations revealed that a short region at the N-terminus comprising amino acids 168–333 can suppress MAD-TIFs without affecting both mitotic ATM kinase and Aurora B-dependent mitotic checkpoint activities. Partial knockdown of TRF2 revealed that such suppressive activity of WRN requires sufficient levels of TRF2. On the other hand, overexpression of TRF2, which can completely suppress MAD-TIFs, failed to do so in WRN-depleted cells, suggesting that WRN enhances the function of TRF2 during mitotic arrest. The N-terminus of WRN harbors four potential Aurora B consensus sequences, and we found that phosphomimetic mutations at S282 abolish the suppressive function of the WRN fragment, suggesting an inhibitory effect of phosphorylation at this site. Our results suggest that the N-terminus of WRN plays a role in preserving the T-loop configuration during mitotic arrest and that WRN cooperates with and supports TRF2 to counteract the cellular pathway that resolves the T-loop during mitotic arrest.

## Results

### WRN non-catalytically suppresses mitotic telomere deprotection

**Figure 1:**
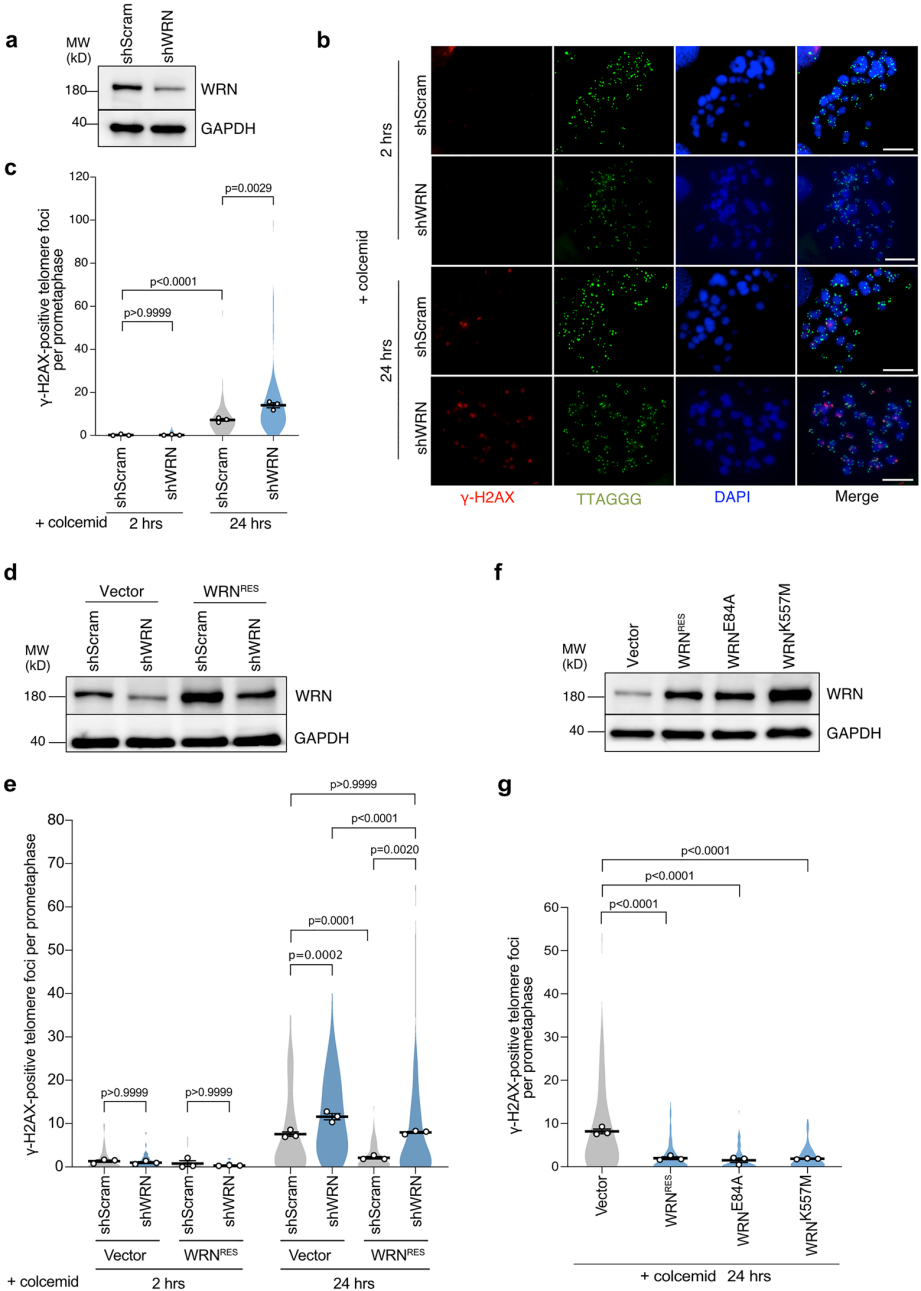
Mitotic telomere deprotection is suppressed by WRN helicase independently of its catalytic activities. (**a**) Immunoblot of WRN in IMR-90 E6E7 hTERT cells transduced with shWRN or shScramble for 5 days. GAPDH serves as a loading control. (**b**) Representative images of meta-TIF assay from WRN knockdown cells after treatment with 100 ng/ml colcemid. The images show DAPI (blue), γ-H2AX (red), and telomere FISH (green). Scale bar, 10 µm. (**c**) Quantification of telomeric signals colocalized with γ-H2AX foci per chromosome spread in indicated conditions. Violin plots illustrate the distribution of all data and averages from three independent experiments (n = 15/experiment for 2 h colcemid; n = 30/experiment for 24 h colcemid; mean ± s.e.m.; Kruskal–Wallis followed by Dunn’s test). (**d**) Immunoblot of WRN in IMR-90 E6E7 hTERT cells expressing exogenous WRN^RES^ or Vector. Cells were transduced with shScramble or shWRN for 5 days before analysis. GAPDH serves as a loading control. (**e**) Quantification of telomeric signals colocalized with γ-H2AX foci in indicated conditions as shown in **c** (n = 15/experiment for 2 h colcemid; n = 30/experiment for 24 h colcemid; mean ± s.e.m.; Kruskal–Wallis followed by Dunn’s test). (**f**) Immunoblot of WRN in cells expressing WRN^RES^ and indicated WRN mutants. Transduced cells were harvested on day 10 post-infection. GAPDH serves as a loading control. (**g**) Quantification of telomeric signals colocalized with γ-H2AX foci in indicated conditions as shown in **c** (n = 30/experiment; mean ± s.e.m.; Kruskal–Wallis followed by Dunn's test). Unprocessed blot images are shown in Supplementary Information files.

WRN helicase unwinds diverse DNA substrates, including D-loops that can form within the protective T-loop structure^26^. To investigate whether WRN is involved in telomere deprotection during prolonged mitotic arrest, we used lentiviral transduction of short hairpin RNA (shRNA) to deplete WRN in telomerase-immortalized IMR-90 fibroblasts expressing HPV oncogenes E6 and E7, which target p53 and Rb tumor suppressors, respectively^27^ (Fig. 1a). Cells efficiently transduced were treated with colcemid for 2 h as a control and 24 h to induce mitotic arrest. Control cells displayed minor colocalization of γ-H2AX signal with the telomere signal on prometaphase/metaphase chromosomes (meta-TIFs) upon 2 h colcemid treatment, while meta-TIFs increased after 24 h of colcemid treatment (Fig. 1b,c), consistent with previous findings^13^. Thus, the meta-TIFs that arose due to prolonged colcemid treatment were defined as mitotic arrest-dependent TIFs (MAD-TIFs), resulting from mitotic telomere deprotection. We found that WRN knockdown does not affect meta-TIFs upon 2 h colcemid but significantly increases the number of MAD-TIFs compared to the shScramble upon 24 h colcemid (Fig. 1c). We confirmed that the expression of a full-length WRN^RES^ carrying silent mutations at shRNA-target sequences restores this exacerbated MAD-TIF phenotype (Fig. 1d,e), as well as recovering growth defect caused by shWRN (Fig. S1a). Moreover, the shScramble cells expressing exogenous WRN^RES^ reduced the number of MAD-TIFs significantly (Fig. 1e). Overall, the number of MAD-TIFs correlates negatively with the protein level of WRN (Fig. 1d,e). The protein level of TRF2 did not increase during mitotic arrest in WRN^RES^-expressing cells (Fig. S1b), suggesting that WRN does not suppress MAD-TIFs by regulating the protein level of TRF2. These results indicate that WRN negatively regulates mitotic telomere deprotection.

It has been shown that mitotic telomere deprotection is a time-dependent event that accumulates during mitotic arrest^13^. Therefore, we addressed whether WRN depletion or exogenous expression affected the mitotic duration upon colcemid exposure, thereby indirectly affecting mitotic telomere deprotection. We performed live-cell imaging of cells treated with colcemid for 72 h (Fig. S1c). As a control, untreated cells were tracked and exhibited an average mitotic duration shorter than 2 h, which is defined as normal. Colcemid treatment induced prolonged mitosis, which reached 20 h on average in shScramble and empty vector cells. Depletion of WRN slightly shortened mitotic duration by an average of 15 h (Fig. S1c), despite WRN-depleted cells possessing increased MAD-TIFs (Fig. 1c,e). Cells expressing exogenous WRN^RES^ showed comparable mitotic duration to the vector control. These results exclude the possibility that suppression of mitotic telomere deprotection by WRN was indirectly caused by modification of mitotic duration.

Since exogenous WRN expression suppressed mitotic telomere deprotection, we aimed to investigate whether this event depends on the WRN enzymatic activities by using two WRN mutants, WRN^E84A^ and WRN^K577M^, which impair 3'-5' exonuclease and helicase activities, respectively^28,29^. We found that both mutants suppressed MAD-TIFs as efficiently as WRN^RES^ (Fig. 1f,g). This data suggests that WRN suppresses mitotic telomere deprotection independently of its enzymatic activities.

### The N-terminus 168–333 aa of WRN is sufficient to suppress mitotic telomere deprotection

**Figure 2:**
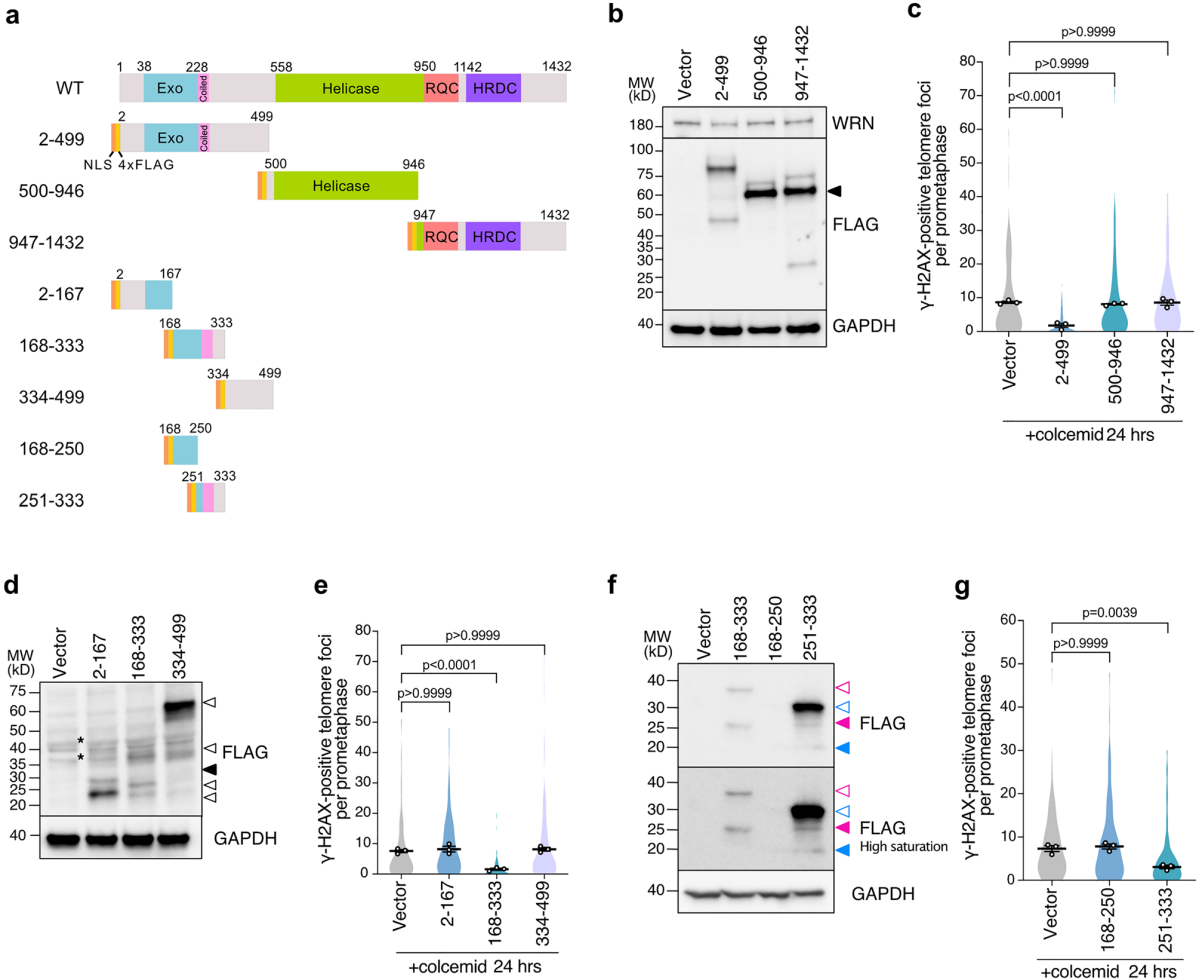
WRN N-terminus coiled-coil region is sufficient to suppress MAD-TIFs. (**a**) Schematic representation of NLS and 4xFLAG tagged WRN fragments and derivative sub-fragments from the N-terminal WRN^2–499^ fragment. Exo, exonuclease domain; Coiled, coiled-coil motif; Helicase, helicase domain; RQC, RecQ C-terminal DNA-binding domain; HRDC, helicase and RNaseD C-terminal domain. (**b**) Immunoblot of endogenous WRN and NLS-4FL-WRN fragments in IMR-90 E6E7 hTERT cells expressing indicated WRN fragments. Transduced cells were analyzed on day 10 post-infection. A black arrowhead indicates bands of the expected size of 60–70 kDa. GAPDH serves as a loading control. (**c**) Quantification of telomeric signals colocalized with γ-H2AX foci in indicated conditions as shown in Fig. 1c (n = 30/experiment; mean ± s.e.m.; Kruskal–Wallis followed by Dunn's test). (**d**) Immunoblot of FLAG-WRN fragments in IMR-90 E6E7 hTERT cells expressing indicated WRN fragments. Transduced cells were harvested on day 12 post-infection. A black arrowhead indicates the expected fragment size (~ 27 kDa). Potential truncation, complex formation, and post-translational modifications are specified with white arrowheads. Asterisks represent unspecific bands from empty Vector. GAPDH serves as a loading control. (**e**) Quantification of telomeric signals colocalized with γ-H2AX foci in indicated conditions as shown in Fig. 1c (n = 30/experiment; mean ± s.e.m.; Kruskal–Wallis followed by Dunn's test). (**f**) Immunoblot of FLAG-WRN fragments in IMR-90 E6E7 hTERT cells expressing indicated WRN fragments. Transduced cells were harvested on day 12 post-infection. Magenta and blue arrowheads indicate the expected size for the WRN168-333 (~ 27 kDa) and the WRN251-333 (~ 17 kDa) fragments, respectively. White arrowheads with colored lines indicate possible post-translational modifications or complex formation for each mutant. (**g**) Quantification of telomeric signals colocalized with γ-H2AX foci in indicated conditions as shown in Fig. 1c (n = 30/experiment; mean ± s.e.m.; Kruskal–Wallis followed by Dunn's test). Unprocessed blot images are shown in Supplementary Information files.

To identify the regions of WRN required for the suppression of mitotic telomere deprotection, three distinct truncated fragments, WRN^2–499^, WRN^500–946^, and WRN^947–1432^, were tagged with NLS and 4xFLAG at N-terminus and expressed in IMR-90 E6E7 hTERT cells (Fig. 2a). We found that all fragments appeared on blots with additional bands, possibly from post-translational modifications (PTMs) of unknown nature or N-terminus multimerization that has been reported to be resistant to SDS denaturation^30^ (Fig. 2b). The higher molecular band of the N-terminal WRN accumulated in mitotic cells compared to interphase cells, suggesting potential PTMs of the fragment during mitotic arrest (Fig. S2a,b). Cell cycle synchronization also revealed that the protein levels of endogenous WRN increased in mitosis, suggesting potential upregulation of WRN during mitosis (Fig. S2b). We found that expression of the NLS-4FL-WRN^2–499^ fragment, but not other fragments, significantly suppressed MAD-TIFs compared to the vector control (Fig. 2c). Live cell analysis revealed no difference in mitotic duration upon colcemid treatment, confirming that the suppressive effect of NLS-4FL-WRN^2–499^ is not due to its effect on mitotic duration (Fig. S2c).

To further explore the specific region of the WRN N-terminus responsible for the suppression of MAD-TIF formation, the WRN^2–499^ fragment was separated into three fragments and expressed in IMR-90 E6E7 hTERT cells (Fig. 2a,d). We found the appearance of additional bands possibly due to PTMs, their cleavage, protein complex formation, or multimerization^30^ (Fig. 2d). Among the three fragments, only NLS-4FL-WRN^168–333^ suppressed MAD-TIFs (Fig. 2d,e, S3a). NLS-4FL-WRN^168–333^ completely abolished MAD-TIFs even in the shWRN background (Fig. S3b), suggesting the higher suppressive potential of this fragment compared to the full-length WRN. This suppressive region contains a coiled-coil domain comprising amino acids 228–333 that mediate the multimerization of WRN^30^. Further cleavage of this region into two fragments revealed that NLS-4FL-WRN^251–333^ was observed as a high-intensity upper-shifted band on SDS-PAGE and that its expression partially suppresses mitotic telomere deprotection (Fig. 2a,f,g). On the other hand, we failed to detect the expression of NLS-4FL-WRN^168–250^ despite the successful separation of the protein for this sample (Fig. 2f, S3c), suggesting this fragment was subject to protein degradation. As a result, an average number of meta-TIFs was comparable to the control, and we could not conclude whether WRN^168–250^ also has a suppressive effect or not. The negative effect of NLS-4FL-WRN^168–333^ expression on MAD-TIFs was also confirmed in fibrosarcoma cell line HT1080 (Fig. S3d–f). From these results, we concluded that the WRN^2–499^ and WRN^168–333^ fragments possess a fully repressive effect on mitotic telomere deprotection (Supplementary Table S1) and decided to use these fragments for subsequent experiments.

### Exogenous WRN expression does not impair ATM or Aurora B activities during mitotic arrest

**Figure 3:**
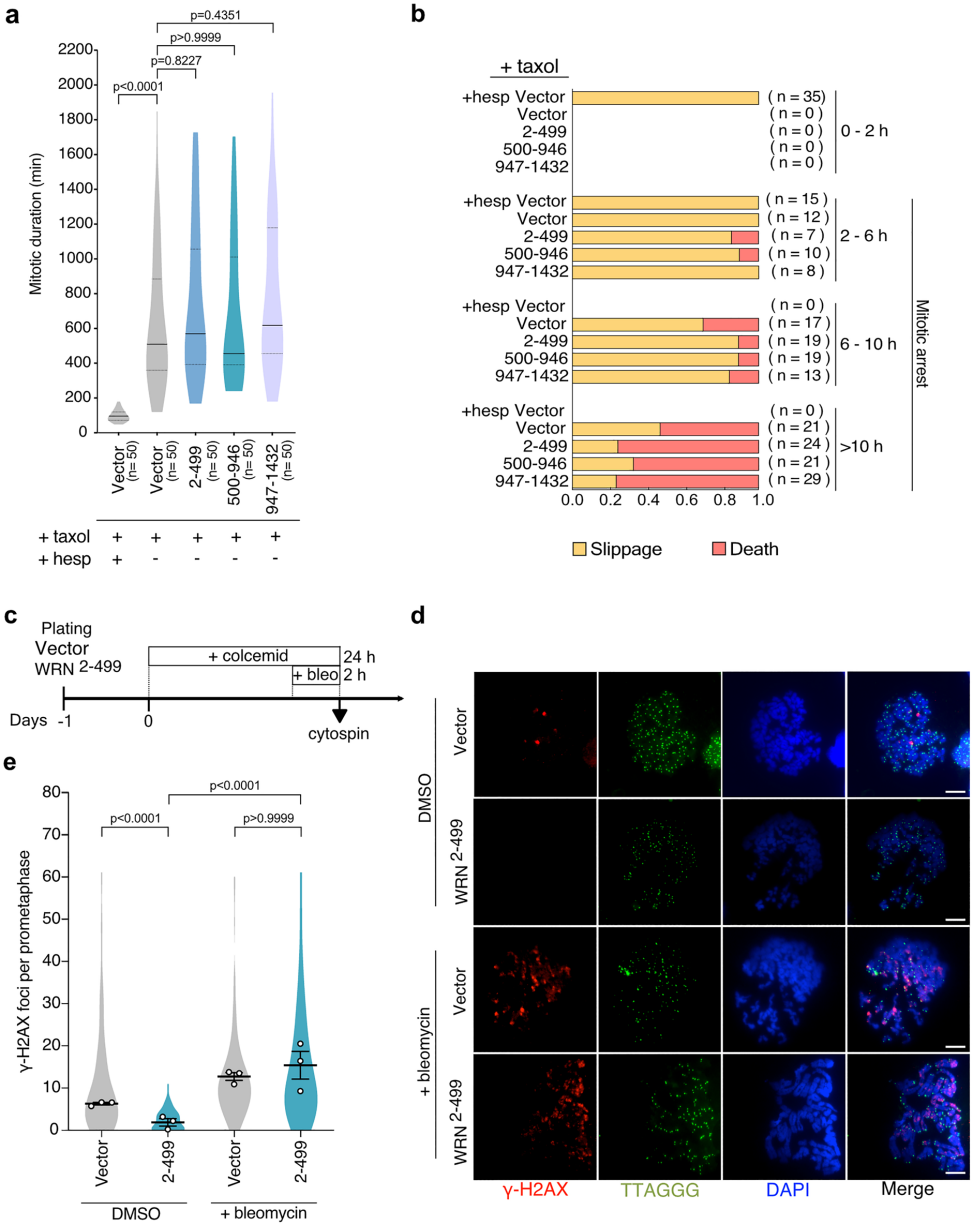
WRN N-terminus does not perturb mitotic Aurora B or ATM kinase activities. (**a**) Distribution of mitotic duration in cells expressing indicated WRN fragments (Median, 25th, and 75th percentile; Kruskal–Wallis followed by Dunn's test). Cells were exposed to 500 nM taxol and analyzed by live-cell imaging data. Vector cells were co-treated with 40 nM Hesperadin as a control. (**b**) Ratio of each cell fate after mitosis in indicated cells. Results are separately shown as categorized by the duration of mitotic arrest. Mitosis longer than 2 h was defined as mitotic arrest. (**c**) Schematic of the timing of 100 ng/ml colcemid and 0.2 µg/ml bleomycin treatment in IMR-90 E6E7 hTERT cells expressing NLS-4xFL-WRN^2–499^ fragment. (**d**) Representative images of γ-H2AX foci on mitotic chromosomes in cells treated as in (**c**). Images show DAPI (blue), γ-H2AX (red), and telomere FISH (green). Scale bar, 10 µm. (**e**) Quantification of total γ-H2AX foci on mitotic chromosomes. Violin plots illustrate the distribution of all data and averages from three independent experiments (n = 30/experiment; mean ± s.e.m.; Kruskal–Wallis followed by Dunn's test).

Since Aurora B kinase is required to promote mitotic telomere deprotection^13^, we asked whether WRN could have an inhibitory effect on Aurora B activity. To test this hypothesis, we exploited a report that Aurora B activity is required to activate the SAC machinery in response to microtubule stabilizer taxol^31^. Live-cell imaging revealed that taxol-induced mitotic arrest in vector control cells is almost completely suppressed by low-dose Hesperadin, an Aurora B inhibitor (Fig. 3a). In contrast, cells expressing WRN fragments showed robust mitotic arrest in response to taxol as vector control (Fig. 3a). A rapid mitotic slippage within 2 h of Hesperadin treatment was observed in vector cells, which is a consequence of Aurora B inhibition^31^ (Fig. 3b). However, cells expressing WRN fragments behaved similarly to the untreated vector control (Fig. 3b). These results indicate that Aurora B remains active in NLS-4FL-WRN^2–499^-expressing cells. Therefore, the lack of MAD-TIF formation is not due to the inhibition of Aurora B activity or its alterations in mitotic response.

WRN has been shown to regulate ATM activation upon DSBs in cells challenged by replication fork collapse during S-phase^32^. Since ATM is required for telomeric γ-H2AX foci formation during mitotic arrest^13^, we questioned whether the N-terminal WRN fragment impairs ATM activation during mitosis. Cells expressing NLS-4FL-WRN^2–499^ fragment were arrested in mitosis by colcemid for 22 h and then exposed to 0.2 µg/ml bleomycin, a DNA damage inducer, for 2 h to induce DNA double-strand breaks into mitotic chromosomes (Fig. 3c). Whereas NLS-4FL-WRN^2–499^ suppressed γ-H2AX foci formation in the absence of bleomycin, a significant increase of γ-H2AX foci on mitotic chromosomes was observed when exposed to bleomycin similarly to control cells (Fig. 3d,e). These results suggest that ATM is still active during mitotic arrest in cells expressing the WRN^2–499^ fragment and that exposure of telomere ends to ATM kinase is suppressed by the N-terminus of WRN during prolonged mitotic arrest.

### WRN supports TRF2 to protect mitotic telomeres

**Figure 4:**
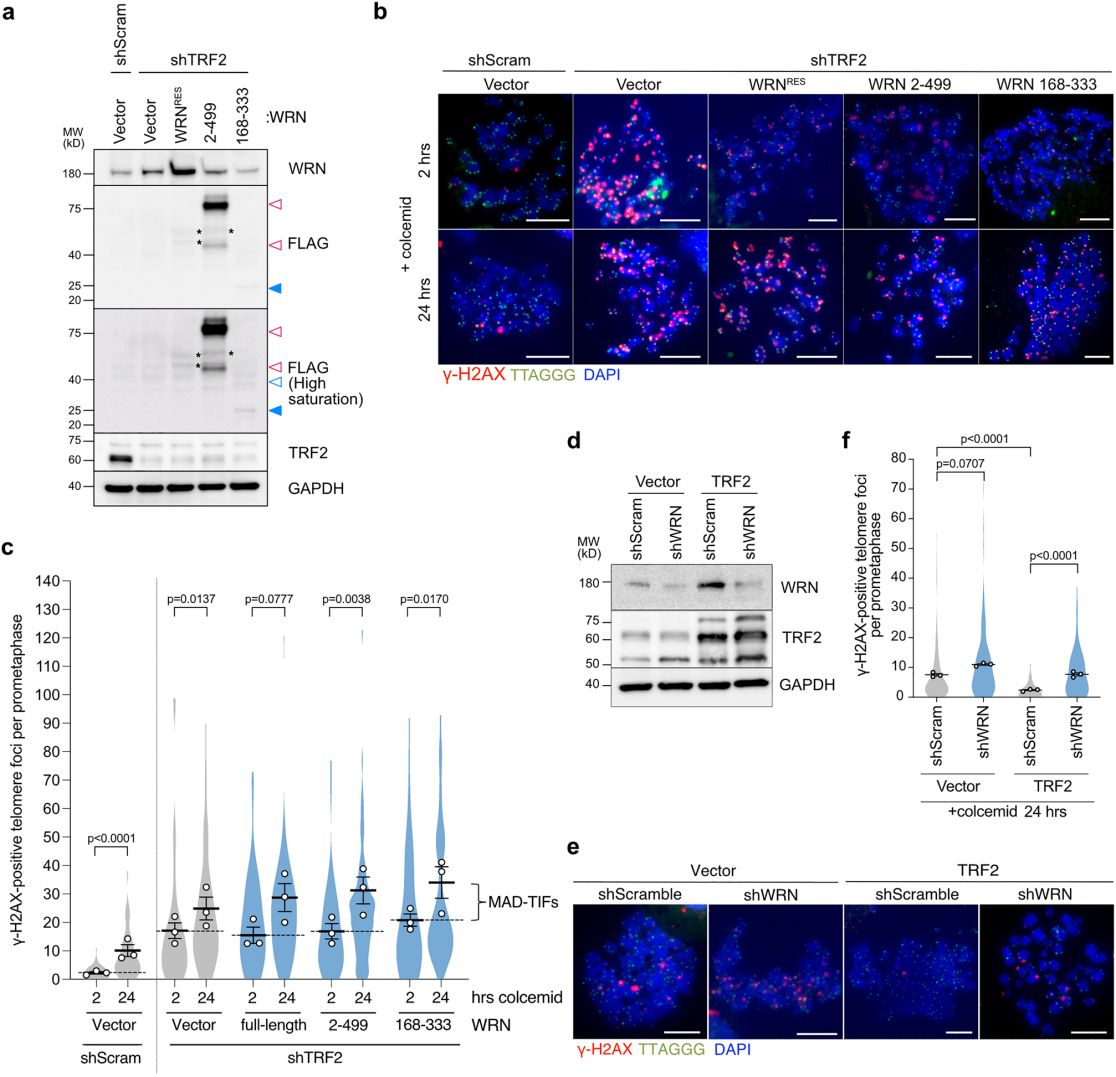
WRN supports TRF2 function to protect mitotic telomeres. (**a**) Immunoblot of endogenous WRN, FLAG-WRN fragments, and TRF2 upon TRF2 depletion in indicated cells. IMR-90 E6E7 hTERT cells expressing indicated WRN fragments were transduced with shTRF2 lentivirus and analyzed on day 7 post-infection. Blue-colored arrowhead indicates the expected size for WRN^168–333^ (~ 27 kDa). White arrowheads (magenta line border for WRN^2–499^ fragment and blue border for WRN^168–333^) indicate possible post-translational modifications or complex formation. GAPDH serves as a loading control. (**b**) Representative images of meta-TIF assay from TRF2 knockdown cells expressing indicated WRN fragments after treatment with 100 ng/ml colcemid. The images show DAPI (blue), γ-H2AX (red), and telomere FISH (green). Scale bar, 10 µm. (**c**) Quantification of telomeric signals colocalized with γ-H2AX foci in indicated conditions as shown in Fig. 1c (n = 15/experiment for 2 h colcemid; n = 30/experiment for 24 h colcemid; mean ± s.e.m.; Mann–Whitney test). Dashed lines discriminate between the average number of TIFs generated in the interphase due to shTRF2 (2 h colcemid) and MAD-TIFs caused by mitotic arrest (24 h colcemid). (**d**) Immunoblot of endogenous WRN and TRF2 in indicated cells. IMR-90 E6E7 hTERT cells expressing exogenous TRF2 were transduced with shWRN lentivirus and analyzed on day 5 post-infection. GAPDH serves as a loading control. (**e**) Representative images of meta-TIF assay in indicated cells from **d** after treatment with 100 ng/ml colcemid for 24 h. The images show DAPI (blue), γ-H2AX (red), and telomere FISH (green). Scale bar, 10 µm. (**f**) Quantification of telomeric signals colocalized with γ-H2AX foci in indicated conditions as shown in Fig. 1c (n = 30/experiment; mean ± s.e.m.; Kruskal–Wallis followed by Dunn's test). Unprocessed blot images are shown in Supplementary Information files.

TRF2 is central in T-loop protection and shows a high binding specificity for the D-loop substrates^33,34^. Since WRN binds to Holliday junctions with high specificity as a tetramer^35^, we asked whether WRN can safeguard mitotic telomeres without the aid of TRF2. For this purpose, we induced telomere deprotection by shRNA-mediated partial depletion of TRF2, which induces TIFs without causing end-to-end telomere fusion^6^, in cells expressing the full-length WRN or WRN N-terminal fragments (Fig. 4a). Insufficient levels of TRF2 generated a higher number of TIFs in interphase cells, which persist until mitosis^6^ (Fig. 4b,c, shTRF2-vector-col2h). When TRF2-depleted cells were arrested in mitosis, an exacerbated number of meta-TIFs was observed, including TIFs derived from interphase and MAD-TIFs^6,14^ (Fig. 4b,c, shTRF2-vector-col24h). In contrast to the previous results that exogenous WRN expression almost completely suppressed MAD-TIFs (Figs. 1 and 2), neither the full-length WRN nor both N-terminal fragments could suppress MAD-TIFs under partial TRF2 knockdown condition (Fig. 4b,c). The results from the 2 h of colcemid treatment also indicate that the number of interphase-TIFs in the absence of TRF2 was not affected by exogenous WRN expression. We next asked if WRN is required to protect mitotic telomeres in cells with the overexpression of TRF2, which has been shown to suppress MAD-TIFs completely^13^. We found that the suppressive effect of TRF2 overexpression is attenuated in WRN-depleted cells (Fig. 4d–f). These results suggest that WRN supports the function of TRF2 when the protein level of TRF2 is sufficient to protect mitotic telomeres.

### The repressive effect of WRN is regulated through putative phosphosites

**Figure 5:**
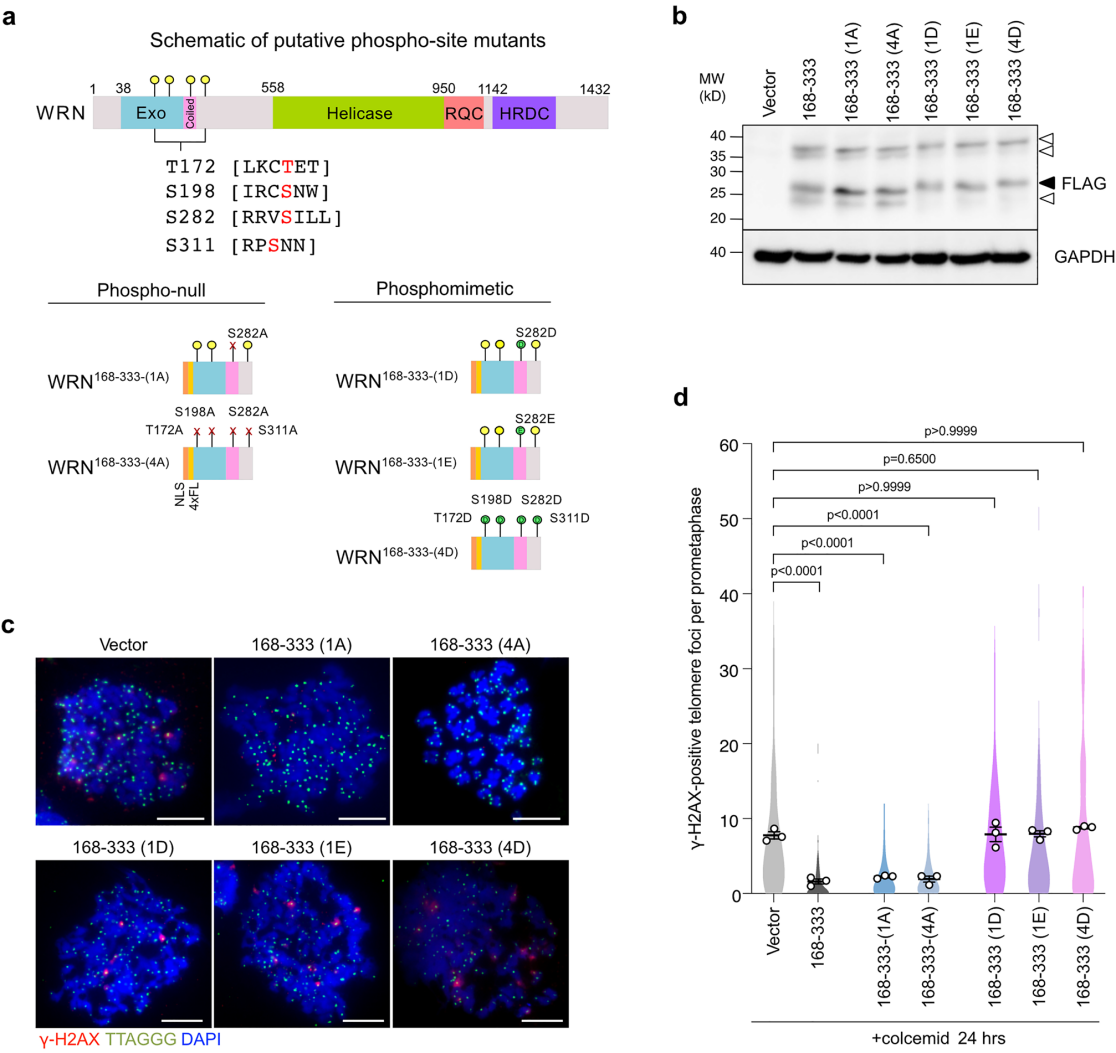
Phosphomimetic mutation at S282 of WRN^168–333^ disrupts its MAD-TIF suppressor effect. (**a**) Schematic representation of four potential Aurora B sites in the 168–333 aa of WRN N-terminus. Sites of alanine and phosphomimetic mutations are indicated below. All mutants contain N-terminal NLS and 4xFLAG tags. (**b**) Immunoblot of FLAG-WRN fragments in indicated cells. Transduced cells were harvested on day 12 post-infection. A black arrowhead indicates the expected band size for all the mutants (~ 27 kDa), and white arrowheads indicate additional bands. (**c**) Representative images of meta-TIF assay in indicated cells after treatment with 100 ng/ml colcemid for 24 h. The images show DAPI (blue), γ-H2AX (red), and telomere FISH (green). Scale bar, 10 µm. (**d**) Quantification of telomeric signals colocalized with γ-H2AX foci in indicated conditions as shown in Fig. 1c (n = 30/experiment; mean ± s.e.m.; Kruskal–Wallis followed by Dunn's test). Unprocessed blot images are shown in Supplementary Information files.

We identified an Aurora B consensus phosphorylation site ([R/K]X[S/T][ϕ], where ϕ is hydrophobic residues)^36^ at position S282 on the WRN^168–333^ fragment and additional three weak putative sites ([R/K]X[S/T]), T172, S198, and S312 (Fig. 5a). Among these sites, Alphafold2 prediction suggests that S282 is in the coiled-coil region (Fig. 5a, S4a). Since Aurora B is required for mitotic telomere deprotection^8,13^, we addressed whether these potential phosphorylation sites are involved in mitotic telomere deprotection by introducing alanine or phosphomimetic aspartic acid and glutamic acid mutations to these sites (Fig. 5a). The alanine mutants exhibited similar band pattern with wild-type WRN fragment, while the phosphomimetic mutants did not show the highest mobility band (Fig. 5b). We found that the alanine-mutated fragments still suppressed MAD-TIFs (Fig. 5c,d). However, all phosphomimetic variants, including S282D and S282E single mutants, failed to suppress MAD-TIFs (Fig. 5c,d). Sequence alignment of WRN orthologs from vertebrate species indicates that S282 is a conserved RX[ST][ϕ] site (Fig. S4b), suggesting the importance of this potential phosphosite. These results imply that the protective effect of the WRN N-terminus is regulated negatively by the phosphorylation at S282.

## Discussion

The T-loop capping structure represses ATM kinase signaling at the natural ends of chromosomes^8^. Telomere-binding protein TRF2 is central to regulating T-loop maintenance during the cell cycle^33^. Cumulative evidence indicates that non-telomeric proteins also play essential roles in T-loop maintenance, especially during the S-phase of the cell cycle^21,37^. Loss of the T-loop in cells undergoing mitotic arrest suggests that non-telomeric proteins also participate in T-loop maintenance at different cell cycle stages and cellular conditions.

In the present study, we showed the role of WRN helicase in regulating mitotic telomere deprotection. Our results indicate that the number of MAD-TIFs inversely correlates with the WRN protein level (Fig. 1d,e). Interestingly, this suppressive effect of WRN is independent of its exonuclease and helicase activities (Fig. 1f,g). Truncation of WRN allowed us to identify that the N-terminal amino acids 168–333 possess a fully repressive effect and that amino acids 251–333 are sufficient to suppress MAD-TIFs partially (Fig. 2). Amino acids 228–333 have been shown to contain a coiled-coil region required for multimerization of WRN^30,38^. The WRN fragments containing this domain showed a higher band shift on SDS-PAGE (Fig. 2b,d,f, Table S1). This band shift could be caused by resistance to SDS-induced denaturation, as reported in WRN fragments containing this domain^30^, although the band size does not agree with multimer sizes (Table S1). The multimerization domain of WRN has been linked to the processivity of exonuclease and efficient strand exchange activities^30,38^. In terms of activity, however, our results strongly argue that the mitotic effect of the N-terminus does not require the catalytic property of WRN. The non-enzymatic function of WRN was also reported in the protection of nascent strands upon replication stress, although the multimerization domain is dispensable for this function^39^. Thus, our finding denotes a novel non-enzymatic function that relies on the coiled-coil domain of WRN to help the maintenance of mitotic telomere caps.

Increasing evidence has shown the effectiveness of WRN in targeting and resolving several DNA substrates found at telomeres, such as G-quadruplex and D-loops^39,40^. However, our results suggest that the D-loop, one of the proposed structures at the T-loop junction, is not resolved by WRN during mitotic arrest. This result may suggest that WRN is enzymatically inactive during mitosis. In another scenario, WRN is still active, but the T-loop may not possess a substrate for WRN. Griffith's group proposed that T-loops could form through the transcription of telomeres, a process that promotes the invasion of blunt-ended telomeric DNA into the transcription bubble^40,41^. As a result, stable junctions, such as three-way forks and Holliday junctions, are generated inside the T-loop, which might not be favorable substrates for WRN.

We found that the protective effect of the WRN coiled-coil domain requires TRF2 residence at mitotic telomeres (Fig. 4). TRF2 has been proposed to be necessary and sufficient for T-loop formation, possibly owing to its ability to promote strand-invasion of the telomeric 3' overhang^4,33,42^. However, our results suggest that TRF2 alone is insufficient to protect T-loop under specific cellular conditions, such as a mitotic arrest. The excessive MAD-TIFs observed in WRN knockdown cells, as well as the failure of TRF2 overexpression in suppressing MAD-TIFs in WRN-depleted cells, supports this notion (Figs. 1a–c and 4d–f). Therefore, our study uncovers the involvement of non-telomeric protein to support TRF2 ability to protect T-loop in mitotically arrested cells. Electron microscopy visualized aggregations of WRN with DNA molecules containing Holliday junctions, a similar substrate to the D-loop junction in telomeres^35^. However, since the WRN^168–333^ fragment lacks a DNA-binding RQC motif, it is less likely that the protective function of WRN relies on a direct DNA binding activity. We thus speculate that WRN affects other protein functions through protein–protein interaction, as discussed below.

Amino acid substitution mimicking phosphorylation on four putative Aurora B target residues and the single conserved S282 residue impaired the suppressive activity of the WRN^168–333^ fragment, whereas alanine mutants still prevented MAD-TIF formation (Fig. 5). Phosphoproteomic analysis identified phosphorylation at S282 in cycling cells^43,44^, which, together with our results, suggest that WRN repressive effect might be regulated by upstream kinases, such as Aurora B kinase. In some proteins, single phosphorylation modifies the α-helical conformation^45^. Since coiled-coils exhibiting hydrophobic side chains can aggregate with other helical strands of self or distinct proteins, side chain phosphorylation or insertion of a negatively charged residue can disrupt protein multimerization, aggregation, and localization^46,47^. Indeed, phosphorylations on α-helices strongly impair the formation of protein aggregates and fibrils due to disruption of the nucleation process^47,48^. Thus, we infer that the WRN^168–333^ domain suppresses MAD-TIF formation by coiled-coil formation between other proteins with α-helices. A potential target of WRN may reside in a pathway that removes TRF2 from mitotic telomeres^13^. Expression of the NLS-4FL-WRN^168–333^ fragment has a more potent suppressive effect compared to the full-length WRN^RES^ in WRN-depleted cells (Fig. 1e, S3b), implying that other WRN domains affect the coiled-coil property or its PTMs. Overall, although further studies are needed to identify a kinase responsible for S282 and to uncover the underlying molecular mechanism, our results using diverse fragments of the N-terminal WRN suggest that the alpha-helix structure of the N-terminus and its PTMs play a role in protecting mitotic telomeres.

Here we identified a novel non-enzymatic function of the WRN N-terminus that may support T-loop maintenance by TRF2 during prolonged mitotic arrest. Our study provides new insights into RecQ helicase functions in mitosis apart from their well-identified catalytic roles in DNA repair and telomere maintenance during the S-phase. The results also raise the possibility that other RecQ helicases might have active roles in mitotic telomere deprotection, an event involved in cell death during telomere crisis and mitotic drug treatment^13,14,16^. Therefore, elucidating the mechanism of mitotic telomere deprotection will provide valuable knowledge to understand the role of telomeres and their regulating factors in mitosis.

## Materials and methods

### Cell culture

IMR-90 E6E7 hTERT cell line was produced through the infection of normal diploid human fibroblasts cells (IMR-90) by retrovirus carrying HPV16 E6 and E7 oncoproteins (pLXSN3-16E6E7)^49^ and immortalized through human telomerase (hTERT) expression by retrovirus carrying wild-type hTert (pWZL-hTERT). Cells were grown in Dulbecco's Modified Eagle Medium (DMEM) supplemented with 10% fetal bovine serum (FBS), 200 mM L-glutamine, 7.5% NaHCO_3_, 100 U/ml penicillin, streptomycin, and 5 μg/ml Plasmocin (InvivoGen) and maintained at 37 °C in 7.5% CO2 and 3% O2.

### Plasmid construction

Truncation and point mutants of WRN were generated by conventional PCR mutagenesis and confirmed by sequencing. All plasmids used in this study are listed in Table S2.

### Lentiviral packaging and transduction

Lentivirus production was performed by transfection of a transfer plasmid with packaging and envelope plasmids gifted from Didier Trono (Addgene plasmid #12260) and Bob Weinberg (Addgene plasmid #8454), respectively, into HEK293FT cells. Media was replaced 24 h post-transfection, and infectious viral supernatants were collected twice through filtration (0.45 µm pore, 25 mm, technolabsc inc.) after 24 and 48 h. Cells were infected with the virus supernatant complemented with 8 μg/ml polybrene for 48 h. Cells stably expressing WRN variants or TRF2 were selected using 10 μg/ml S Blasticidin (Funakoshi) for at least five days. Transduced WRN- and TRF2-expressing cells were used 12 to 15 days post-infection for downstream experiments. For WRN and TRF2 knockdown, cells were infected with lentiviruses produced from pLKO.1-shWRN-2 (pMTH218) containing the shRNA target sequence 5′-CCTGTTTATGTAGGCAAGATT-3′ (TRCN0000004902) or pLKO.1-shTRF2-F (pMTH285) containing 5′-GCGCATGACAATAAGCAGATT-3′ (TRCN0000004811) sequence^6^. Two days post-infection, the selection was carried out by adding 1 μg/ml puromycin (ChemCruz) to cell culture for three days before the experimental procedure.

### Cell growth assay

Transduced vector and WRN^RES^ cells were infected with lentivirus containing shScramble and shWRN sequences for 24 h and selected with 1 μg/ml puromycin. After 3 days of selection, cells were collected and an initial number of 20,000 cells were re-plated in individual wells of a 24-well plate with a working volume of 500 μl of fresh medium. Cells were trypsinized and counted at day 2 and 4 in culture using an automated cell counter (DeNovix CellDrop BF).

### Immunofluorescence and telomere FISH on metaphase spreads

The meta-TIF analysis was performed as described previously^50^. IMR-90 E6E7 hTERT cells were subcultured into a fresh medium and incubated at 37 °C for 24 h. Subsequently, the cells were exposed to 100 ng/mL colcemid for 2 or 24 h to accumulate mitotic cells. For DNA damage induction experiments, 0.2 ug/ml bleomycin was added 2 h before the 24 h colcemid treatment was completed. Cells were collected by cytocentrifugation at 12,000 rpm for 3 min, fixed in 4% formaldehyde solution, and suspended in 0.2% hypotonic KCl solution at RT for 10 min, followed by centrifugation onto glass slides. Metaphase chromosome spreads were fixed in 4% formaldehyde at RT for 10 min and permeabilized with KCM buffer (120 mM KCl, 20 mM NaCl, 10 mM Tris pH 7.5, 0.1% Triton) for 15 min and blocked in ABDIL buffer (150 mM NaCl, 20 mM Tris pH7.4, 0.1% Triton X-100, 2% BSA, 0.2% Fish Gelatin) with 100 μg/mL RNase A at 37 °C for 10 min. Samples were incubated with mouse anti-γ-H2AX p-Ser139 antibody (613,402 Clone 2F3, Biolegend) at 1:200 dilution in ABDIL buffer. Following the staining with the secondary antibody Alexa-568-conjugated anti-mouse (A11031, Invitrogen) at 1:10,000 dilution in 1X TNT, samples were fixed in 4% formaldehyde. Telomere staining was performed by denaturing the slides for 8 min at 80 °C with Tel-C FAM-OO-(CCCTTAA)3 PNA probe (Panagene). After denaturation, hybridization was continued overnight at room temperature in a wet chamber. Slides were washed and dehydrated in ethanol series [70%, 95%, 100% (vol/vol)] and briefly air-dried before DNA was counterstained and mounted with Vectashield PLUS Antifade mounting medium with DAPI (H-2000, Vector Laboratories). Equipment and settings: Images of metaphase spreads were taken with a 100 × objective lens (PlanApo/1.45-NA oil) on a BZ-X710 fluorescence microscope (KEYENCE) and analyzed by automated counting with Hybrid Cell Count and Macro Cell Count software modules (KEYENCE). Outlier values in the data sets were excluded from the analysis.

### Cell cycle synchronization

To synchronize cells, a double-thymidine block was used. The cells were incubated with 2 mM thymidine for 14 h, washed three times with 1xPBS, and then cultured for an additional 10 h in fresh media. They were then treated with 2 mM thymidine for another 14 h and released into fresh media after three more washes with 1xPBS (0 h post-release). Colcemid (100 ng/mL) was added to the medium 6 h later, and mitotic cells were collected by shake-off at 8 and 24 h post-release (2 and 18 h after colcemid treatment), and used for downstream analysis.

### Immunoblotting and immunoprecipitation

Cells were lysed in a lysis buffer complemented with 1X protease inhibitor (ROCHE) and 1X phosphatase inhibitor (ROCHE). Centrifugation was performed for 10 min at 12,000 rpm, and debris was removed. Protein concentration was measured by spectrometry using a Bio-Rad Protein Assay Dye Reagent (5000006JA, Bio-Rad Laboratories). Approximately 50 μg of cell lysate was fractionated by 4–20% Mini-PROTEAN TGX precast gels (BioRad) and transferred onto the PVDF membrane (Millipore). For CBB staining, the gel was incubated for 1 h with CBB Stain One (Nacalai), followed by washes with deionized water. Before immunoblotting, the membrane was blocked for 20 min at RT with Blocking Buffer (Nacalai). Primary antibodies: rabbit anti-WRN (ab124673, Abcam; 1:500 dilution), rabbit anti-TRF2 (NB110-57130SS, Novus Biologicals; 1:1000 dilution), mouse anti-GAPDH (MAB374, Millipore; 1:5000 dilution), and mouse anti-FLAG (F1804, Sigma; 1:1000 dilution). Secondary antibodies: HPR-linked anti-mouse (NA931, GE Healthcare; 1:10,000 dilution) or anti-rabbit (2074, Cell Signaling; 1:10,000 dilution). Membranes were cut to adequate size before the primary antibody, so multiple different blot images were taken from the same membrane. Antibodies on the membrane were detected by ECL reaction and imaged by LAS-3000 (Fuji). Exposure time and signal intensity were adjusted during image acquisition. No digital processing except cropping was performed on the image data. Original blotting images are found in Supplementary Information.

### Live-cell imaging

Cells were seeded in a 48-well plate 24 h before imaging. One hour before imaging, cells were treated with 100 ng/mL colcemid, 500 nM taxol, and 40 nM Hesperadin. Equipment and settings: Time-lapse imaging was then carried out using a microscope incubator system (Tokai Hit) that maintains cells under controlled conditions at 37 °C, 5% CO_2_ for 60 to 72 h on a BZ-X710 microscope. Observations were performed using a 10 × objective (Plan Apo 0.45 NA), and z-stack sections were set to capture approximately 0.7 µm thick optical sections. Mitotic duration and cell fate were manually determined, based on cell morphologies, from the first frame of mitotic entry (*i.e.,* a sign of nuclear envelope breakdown or cell rounding) until the end of the phase (*i.e*., a sign of cytokinesis, nuclear blebbing, or cell flattening). ImageJ and QuickTime Player softwares were used to analyze movie files.

### Protein structure prediction and sequence alignment

Protein structure prediction of the WRN^168–333^ fragment was generated with the ColabFold interface to the AlphaFold 2 pipeline on the Colab platform (AlphaFold2.ipynb)^51,52^. Program config, input sequence, and coordinate files are provided in the Supplementary file. For protein sequence alignment, protein sequences obtained from BLAST were aligned using multiple sequence comparisons by log expectation (MUSCLE) and colored by Clustal X in SnapGene software (GSL Biotech)^53^. Accession numbers for WRN homologous are available in Table S3.

### Statistical analysis

All statistical analyses and graphs were performed using GraphPad Prism software (version 9.0). We did not assume Gaussian distribution and performed two-tailed unpaired non-parametric tests for all quantitative data in this study. Mann–Whitney test was used to compare two samples, while Kruskal–Wallis followed by Dunn’s test was used to compare multiple samples. We considered *p*-value less than 0.05 as statistically significant. Data for the meta-TIF analysis was obtained blindly by our lab technicians.

## Supplementary Information

Supplementary Information.

## Data Availability

All data are archived at Kyoto University and available from the corresponding author upon reasonable request.
