# Supplementary material for: A non-catalytic N-terminus domain of WRN prevents mitotic telomere deprotection
Source: Sci Rep. 2023 Jan 12;13:645. doi: 10.1038/s41598-023-27598-0 (PMC9837040; doi:10.1038/s41598-023-27598-0)

Supplementary Materials

A non-catalytic N-terminus domain of WRN prevents mitotic telomere deprotection

Diana Romero-Zamora[1,2], Makoto T. Hayashi[2,3]*

[1]Graduate School of Biostudies, Kyoto University, Yoshida-konoe, Sakyo, Kyoto, 606-8501 Japan; [2] IFOM-KU Joint Research Laboratory, Graduate School of Medicine, Kyoto University, Yoshida-konoe, Sakyo, Kyoto, 606-8501 Japan; [3]IFOM ETS, the AIRC Institute of Molecular Oncology, via Adamello 16, 20139 Milan, Italy

Content:

Supplementary Figures S1, S2, S3, S4

Supplementary Tables S1, S2, S3

Supplementary Information, Original uncropped blotting images for Fig. 1, 2, 4, 5, S1, S2, S3

Supplementary File, AlphaFold2 program config, input sequence, and coordinate data

Figure S1

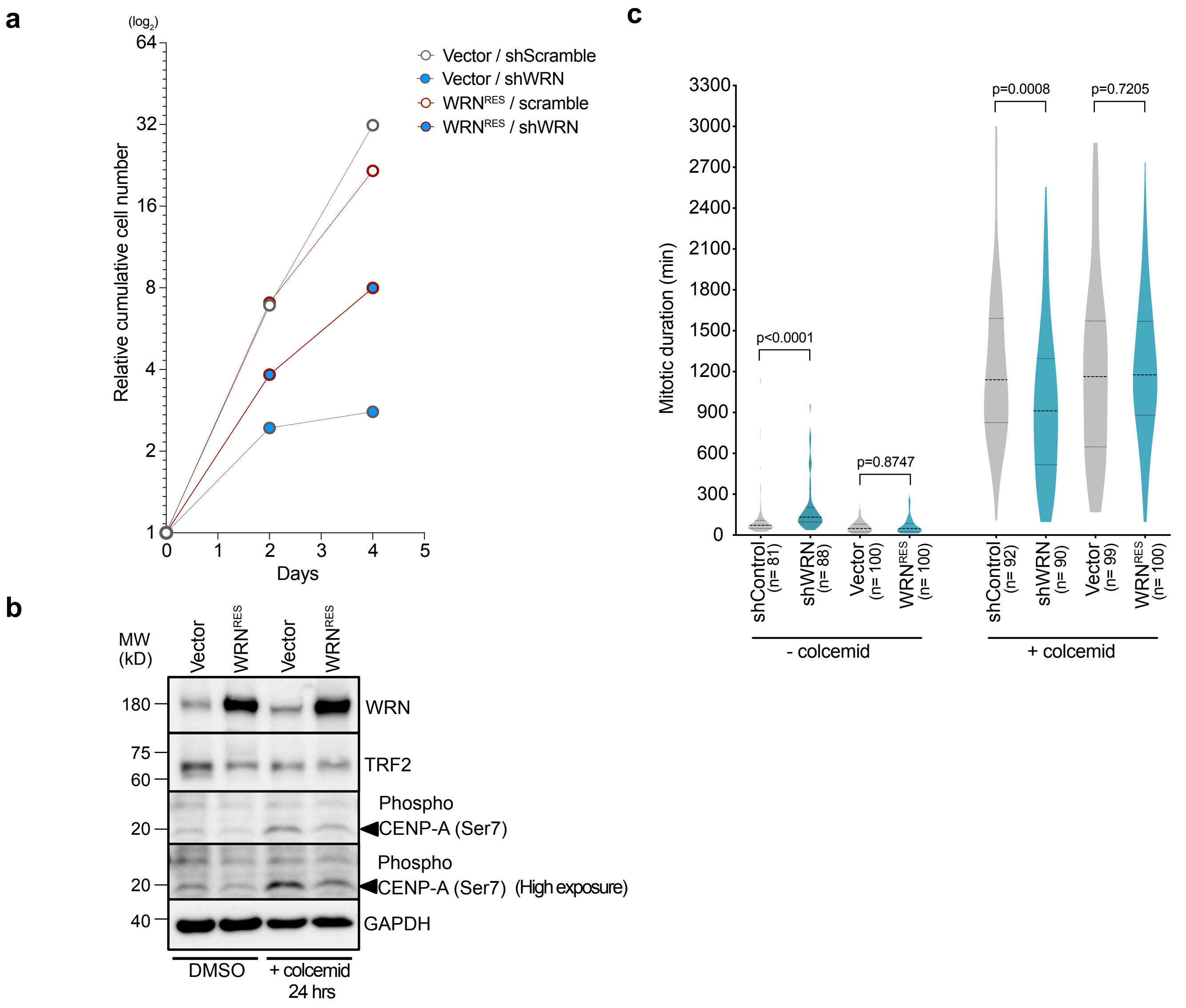

Figure S1. WRN possesses suppressive effect on MAD-TIFs. (**a**) Growth curve of IMR90 hTERT E6E7 cells infected with empty vector or a shRNA-resistant full-length WRN^{RES}, followed by either shScramble or shWRN transduction. The same number of cells were seeded in 24-well plate at day 4 post-infection of shScramble and shWRN and cell number was analyzed at day 2 and 4. (**b**) Immunoblot of TRF2 in cells expressing exogenous WRN after 24 hrs exposure to 100 ng/ml colcemid. Phospho CENP-A (Ser7) and GAPDH serves as a mitotic marker and a loading control, respectively. (**c**) Distribution of mitotic duration in cells expressing shWRN and WRN^{RES} (Median, 25th and 75th percentile; Mann-Whitney test). Cells were either mock-treated or exposed to 100 ng/ml colcemid and analyzed by live-cell imaging.

Figure S2

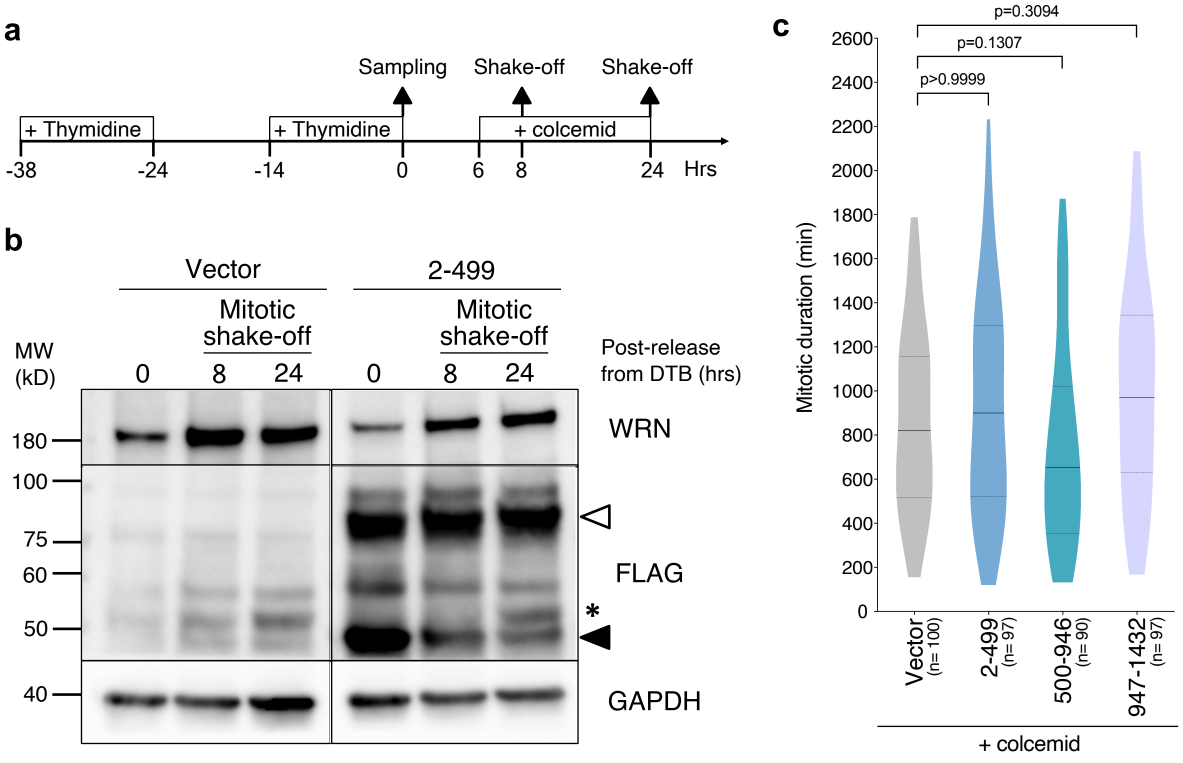

Figure S2. WRN N-terminus is modified upon colcemid treatment without affecting mitotic duration. (**a**) Schematic of the double-thymidine block. Samples for Western blotting were collected at 0, 8, and 24 hrs post the second release. Mitotic cells were collected by shake-off at 8 and 24 hrs. (**b**) Immunoblot of endogenous WRN and the NLS-4FL-WRN2-499 fragment. The expected band size is indicated by a black arrowhead, and potential complex formation or protein modifications are indicated by a white arrowhead. Asterisk denotes unspecific bands. (**c**) Distribution of mitotic duration in cells expressing the indicated WRN fragments (median, 25th, and 75th percentile; Kruskal-Wallis followed by Dunn's test). Cells were treated with 100 ng/mL colcemid and analyzed by live-cell imaging.

Figure S3

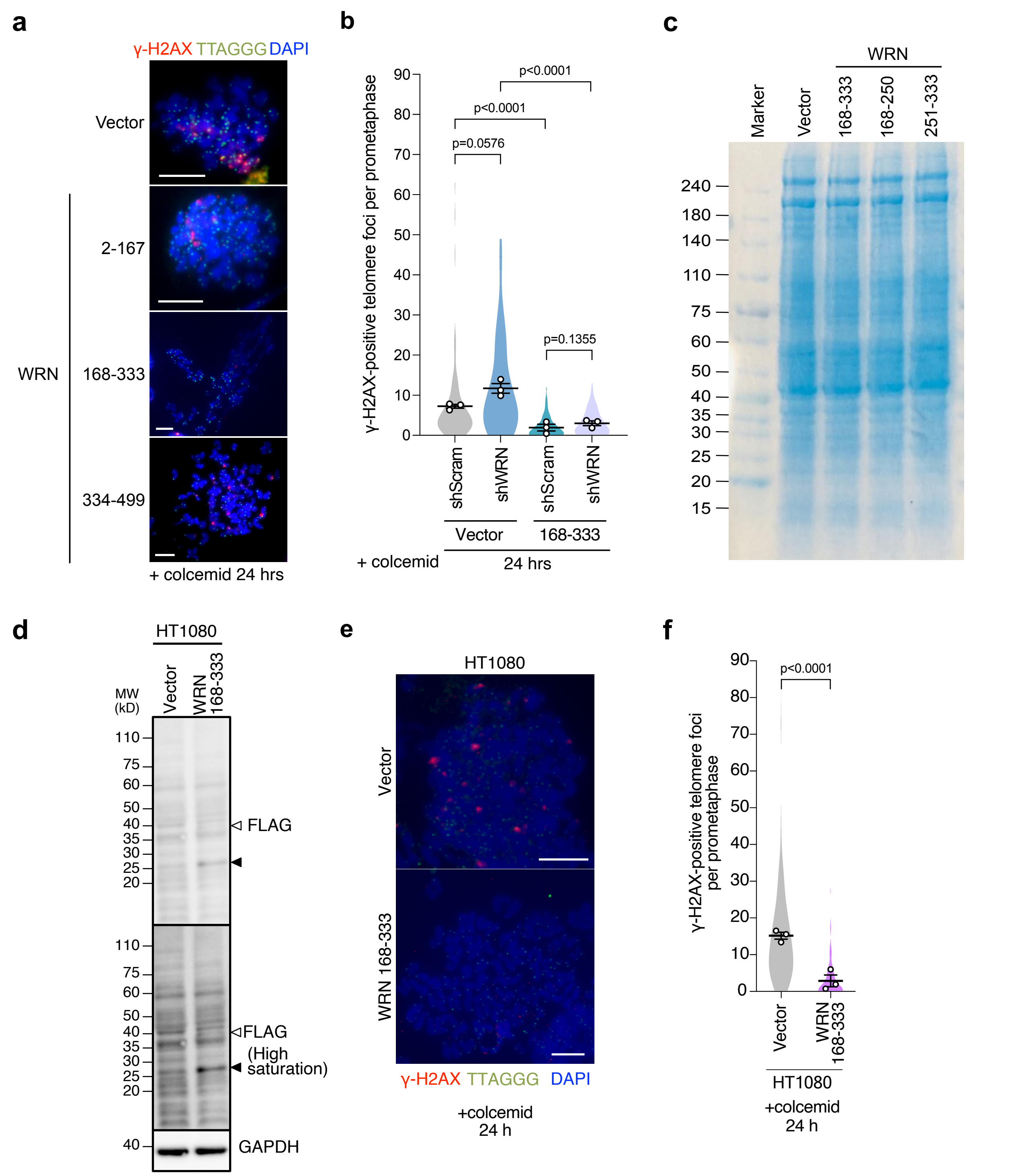

Figure S3. The WRN$^{168\text{-}333}$ fragment containing a coiled-coil suppresses MAD-TIFs. (**a**) Representative images of meta-TIF assay from cells expressing indicated WRN fragments after treatment with 100 ng/ml colcemid for 24 hrs, related to Fig. 2**d** and **e**. Scale bar, 10 μm. (**b**) Quantification of telomeric signals colocalized with γ-H2AX foci in the indicated conditions as shown in Fig. 1**c** (n=30/experiment; mean ± s.e.m.; Kruskal-Wallis followed by Dunn's test). (**c**) Representative SDS-PAGE gel stained with Coomassie Brilliant Blue (CBB) that corresponds to the blot in Fig. 2**f**. (**d**) Immunoblot of FLAG and GAPDH in NLS-4FL-WRN$^{168\text{-}333}$ expressing HT1080 cells. Black and white arrow heads indicate expected size and potentially modified fragment, respectively. (**e**) Representative images of meta-TIF assay in HT1080 cells expressing NLS-4FL-WRN$^{168\text{-}333}$ fragment after treatment with 100 ng/ml colcemid for 24 hrs. The images show DAPI (blue), γ-H2AX (red), and telomere FISH (green). Scale bar, 10 μm. (**f**) Quantification of telomeric signals colocalized with γ-H2AX foci per chromosome spread in NLS-4FL-WRN$^{168\text{-}333}$ expressing HT1080 cells as shown in Fig. 1**c** (n=30/experiment; mean ± s.e.m.; Mann Whitney test).

Figure S4

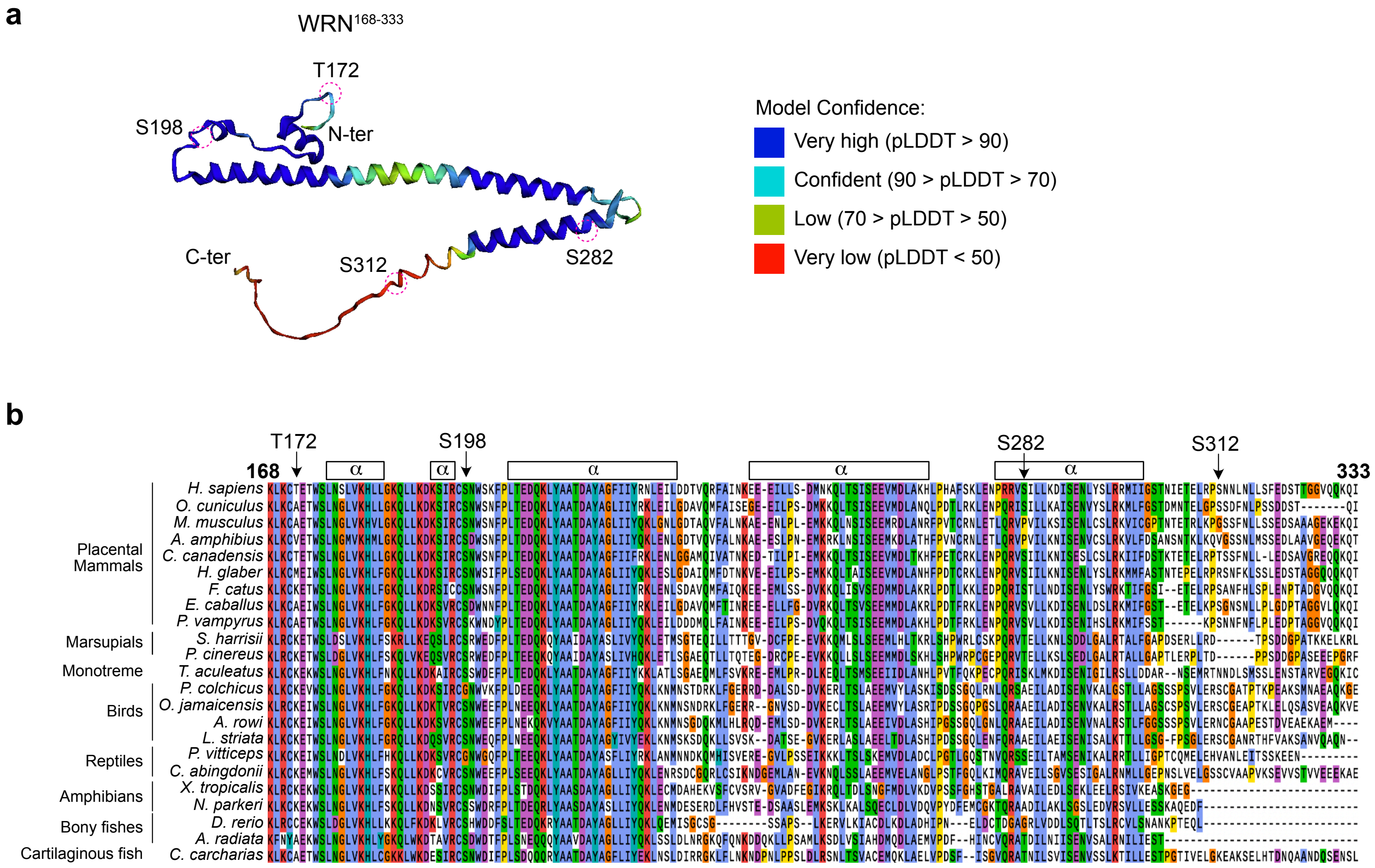

Figure S4. Predicted structure of WRN$^{168\text{-}333}$ and alignment of the protein sequence among vertebrate species. (**a**) A representative structure of the WRN$^{168\text{-}333}$ fragment with a color-coded confidence per-residue (pLDDT) predicted by the AlphaFold2 pipeline. Potential Aurora B target sites are indicated by dashed magenta circles. (**b**) Sequence alignments of human WRN 168-333 aa region with WRN homologs in vertebrate species. Positions of the predicted alpha-helices in **a** are shown above. Arrows indicate putative AURKB phosphorylation sites in the human WRN fragment.

Table S1. Summary of WRN fragments size and phenotypes.

Name	Expected size including NLS-4FL (kD)	Observed band size	MAD-TIF suppression
WRN^{RES}	~180	~180	**YES**
WRN^{2-499}	~69	~48, ~80	**YES**
$WRN^{500-946}$	~62	~62,~70	**NO**
$WRN^{947-1432}$	~67	~27,~63,~74	**NO**
WRN^{2-167}	~27	~23,~27	**NO**
$WRN^{168-333}$	~27	~23,~26,~38	**YES**
$WRN^{334-499}$	~27	~23,~62	**NO**
$WRN^{168-250}$	~17	Not expressed	**NO**
$WRN^{251-333}$	~17	~33	**YES** (partial)
$WRN^{168-333}$-1A	~27	~23,~26,~38	**YES**
$WRN^{168-333}$-4A	~27	~23,~26,~38	**YES**
$WRN^{168-333}$-1D	~27	~26,~38	**NO**
$WRN^{168-333}$-1E	~27	~26,~38	**NO**
$WRN^{168-333}$-4D	~27	~26,~38	**NO**

Table S2. List of plasmids used in this study

Plasmid code (pMTH)	Name	Source
127	pLKO.1-shScramble	Addgene # 1864
218	pLKO.1-shWRN-2 (TRCN0000004902)	Jan Karlseder laboratory
285	pLKO.1-shTRF2-F (TRCN0000004811)	Jan Karlseder laboratory
420	pLenti-blaR-p2a	This study
426	pLenti-blaR-p2a-hWRN_RshRNA	This study
427	pLenti-blaR-p2a-hWRN-E84A_RshRNA	This study
428	pLenti-blaR-p2a-hWRN-K577M_RshRNA	This study
688	psPAX2	Addgene # 12260
689	pCMV-VSV-G	Addgene # 8454
911	pLenti-bla-p2a-TRF2	This study
1174	pLenti-bla-p2a-NLS-4FL-WRN(2-499)	This study
1191	pLenti-bla-p2a-NLS-4FL-WRN(500-946)	This study
1195	pLenti-bla-p2a-NLS-4FL-WRN(947-1432)	This study
1236	pLenti-bla-p2a-NLS-4FL-WRN(2-167)	This study
1241	pLenti-bla-p2a-NLS-4FL-WRN(168-333)	This study
1242	pLenti-bla-p2a-NLS-4FL-WRN(334-499)	This study
1303	pLenti-bla-p2a-NLS-4FL-WRN(168-250)	This study
1304	pLenti-bla-p2a-NLS-4FL-WRN(251-333)	This study
1321	pLenti-bla-p2a-NLS-4FL-WRN(168-333)-4A	This study
1322	pLenti-bla-p2a-NLS-4FL-WRN(168-333)-1A	This study
1351	pLenti-bla-p2a-NLS-4FL-WRN(168-333)-1D	This study
1352	pLenti-bla-p2a-NLS-4FL-WRN(168-333)-1E	This study
1353	pLenti-bla-p2a-NLS-4FL-WRN(168-333)-4D	This study

Table S3. Accession numbers of WRN used in this study

Organism	Accession Number
Homo Sapience	XP_011542941.1
Oryctolagus cuniculus	XP_017194550.1
Mus musculus	XP_017168151.1
Arvicola amphibius	XP_038181556.1
Castor canadensis	XP_020036300.1
Heterocephalus glaber	XP_021092503.1
Felis catus	XP_044911276.1
Equus caballus	XP_023486363.1
Pteropus vampyrus	XP_011371577.1
Sarcophilus harrisii	XP_023361414.1
Phascolarctos cinereus	XP_020863957.1
Tachyglossus aculeatus	XP_038602619.1
Phasianus colchicus	XP_031463388.1
Oxyura jamaicensis	XP_035181081.1
Apteryx rowi	XP_025947442.1
Lonchura striata domestica	XP_021387255.1
Pogona vitticeps	XP_020664644.1
Chelonoidis abingdonii	XP_032625369.1
Xenopus tropicalis	XP_031760210.1
Nanorana parkeri	XP_018426138.1
Danio rerio	XP_021334923.1
Amblyraja radiata	XP_032877913.1
Carcharodon carcharias	XP_041033343.1

Supplementary Information for Fig. 1

Figure 1a

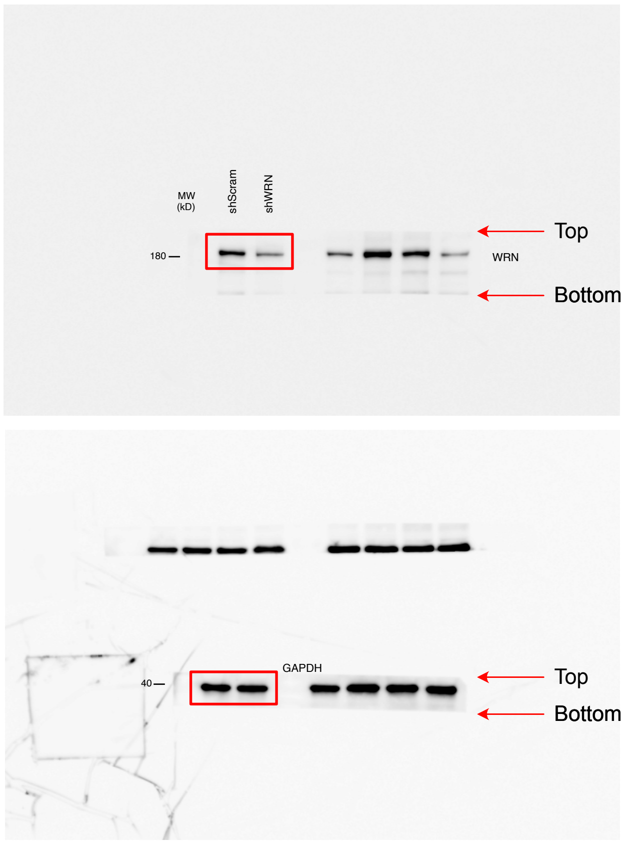

Figure 1f

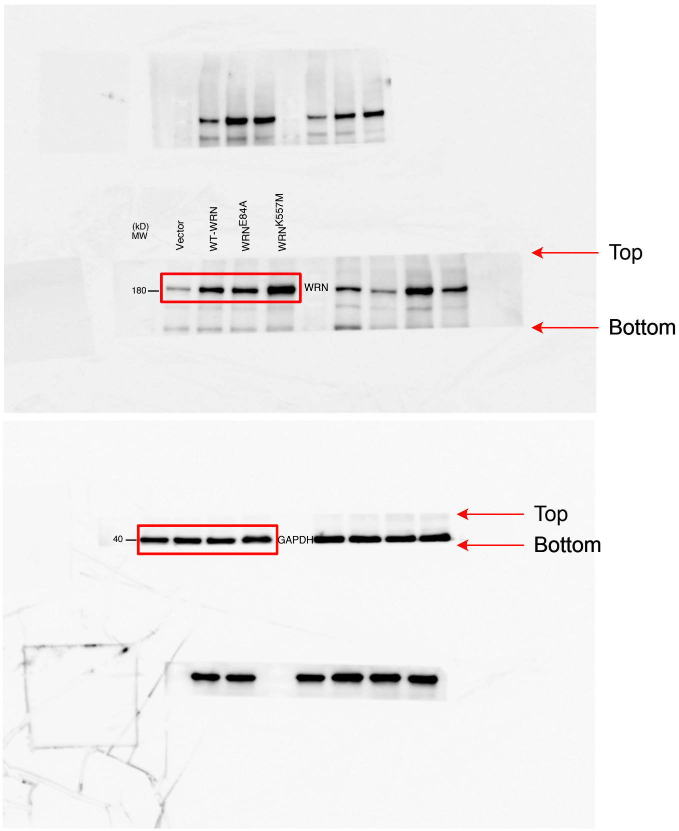

Figure 1d

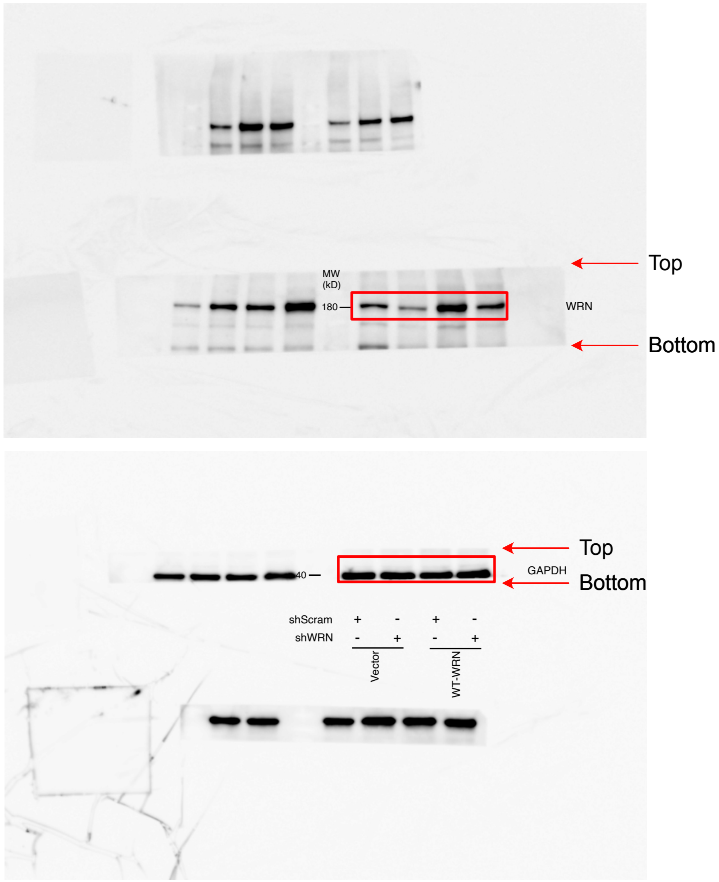

Supplementary Information for Fig. 2

Figure 2b

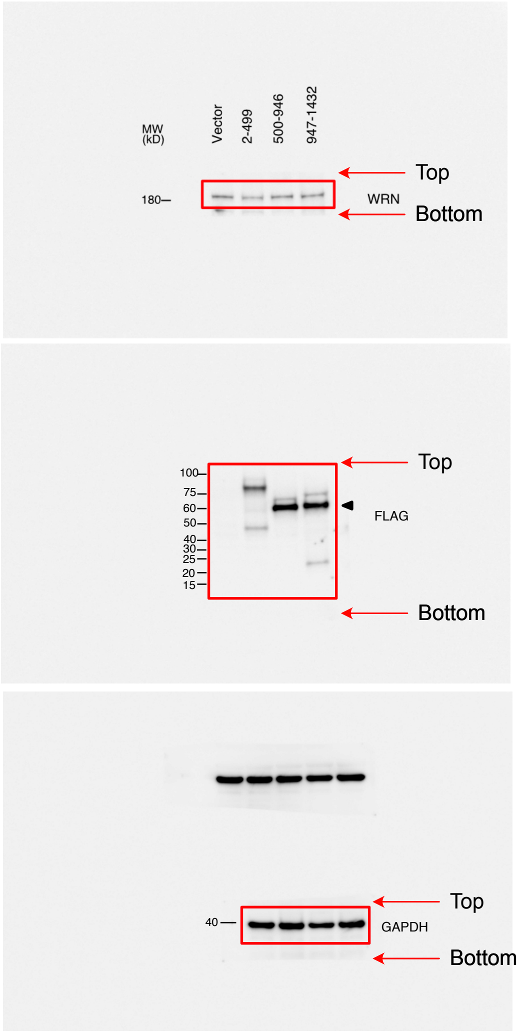

Figure 2d

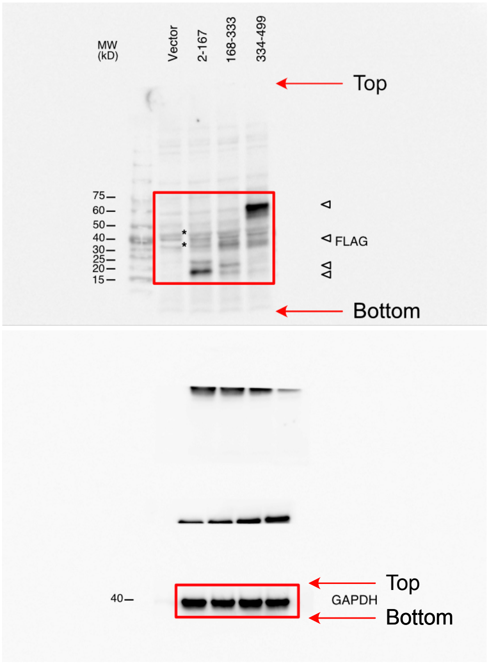

Figure 2f

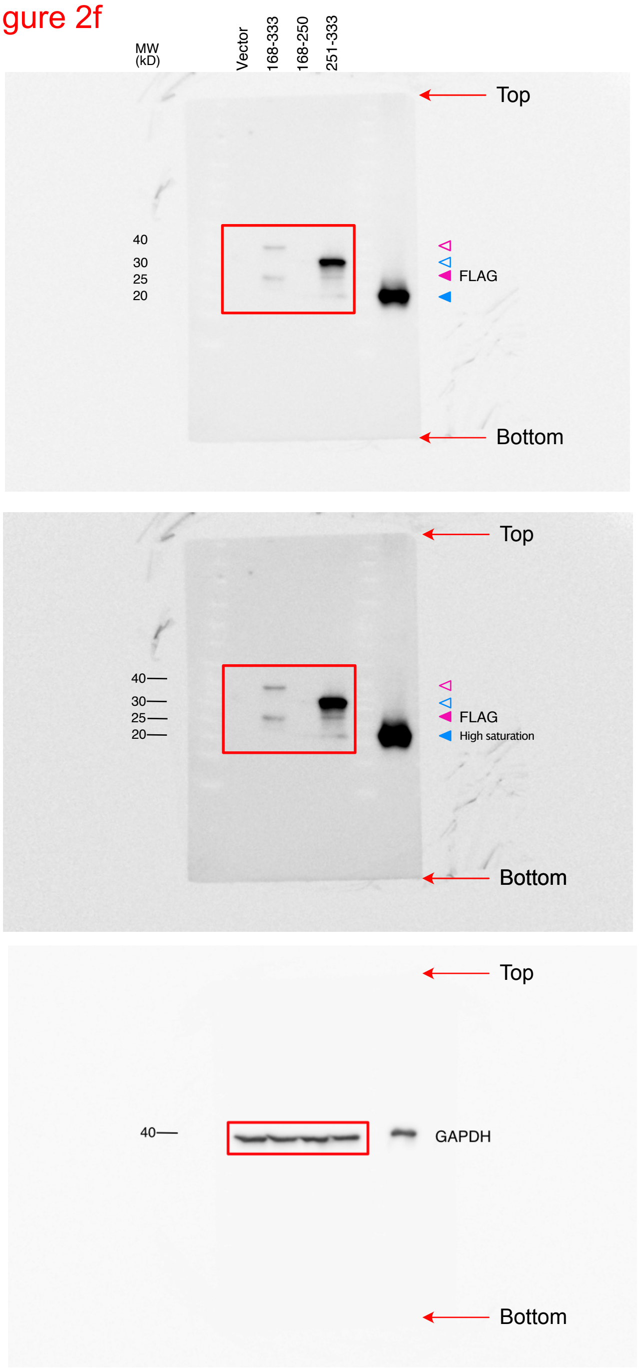

Supplementary Information for Fig. 4

Figure 4a

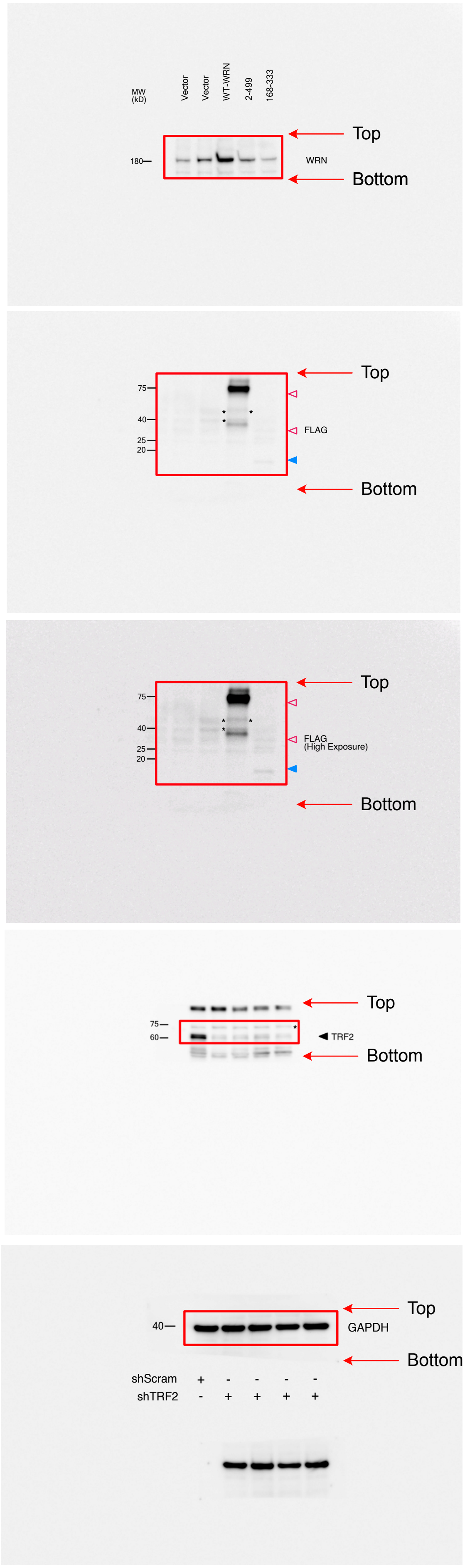

Figure 4d

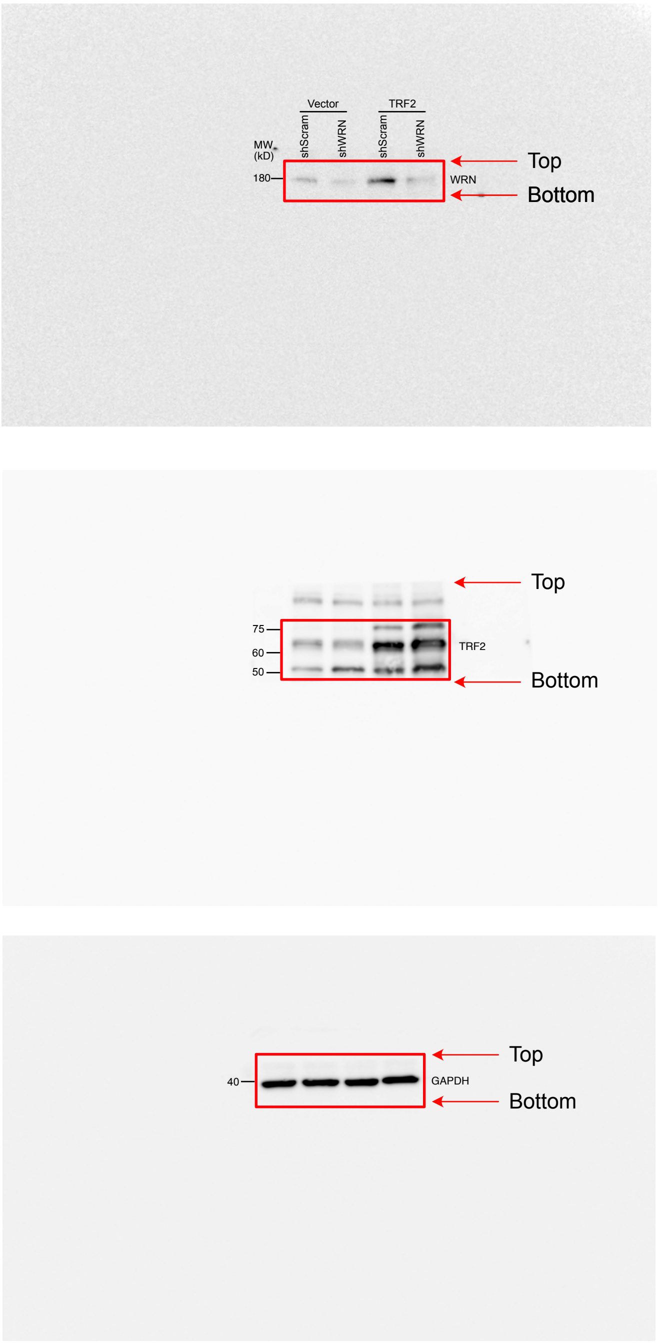

Supplementary Information for Fig. 5

Figure 5b

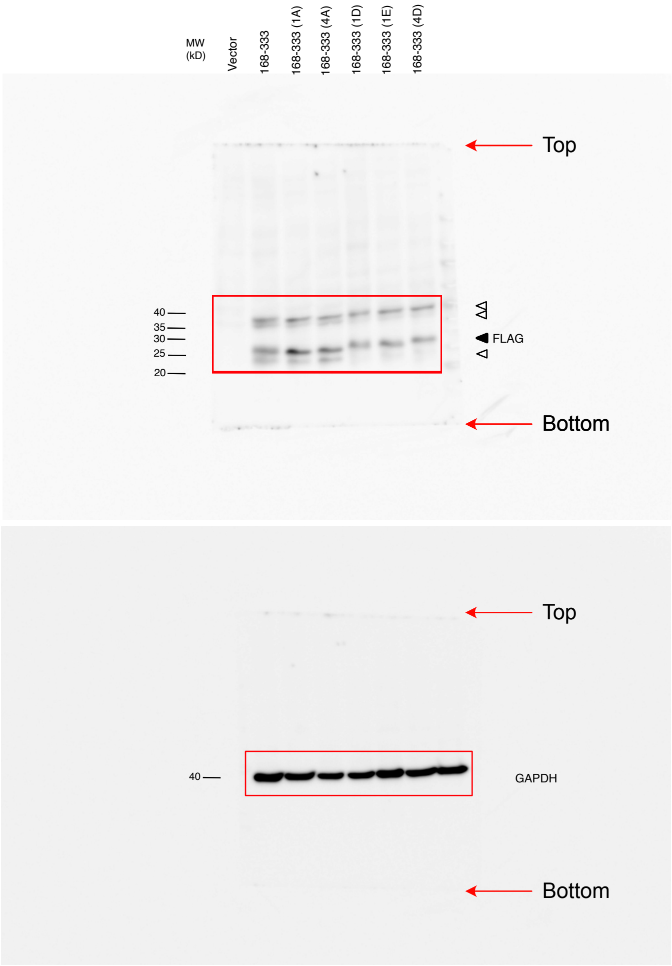

Supplementary Information for Fig. S1

Figure S1b

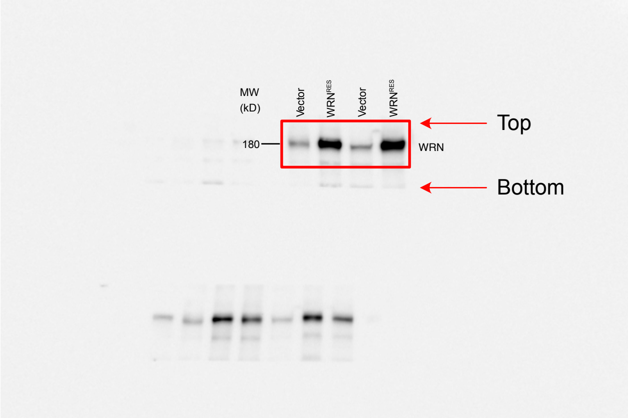

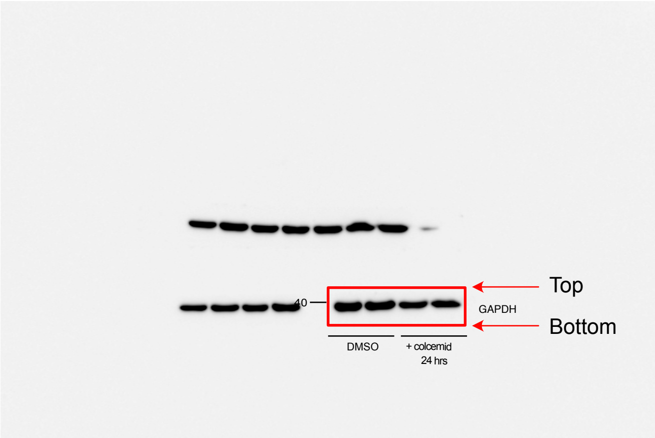

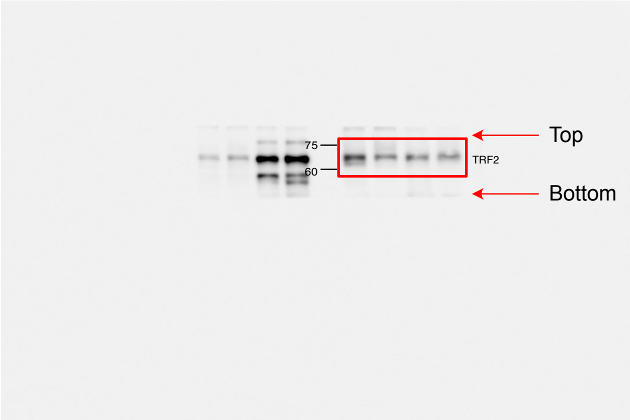

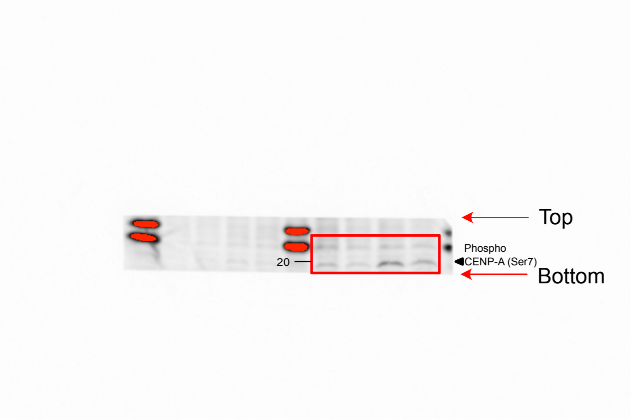

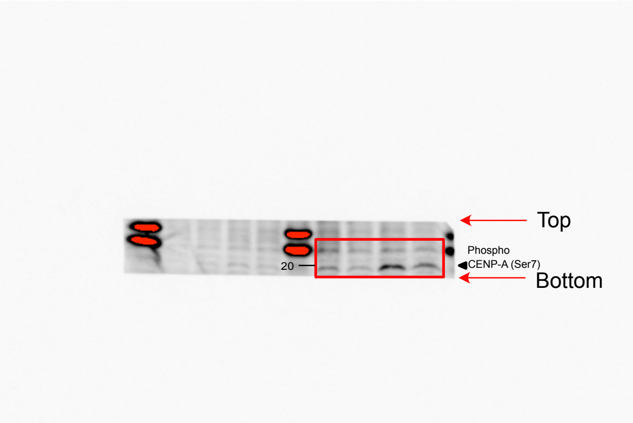

Supplementary Information for Fig. S2

Figure S2b

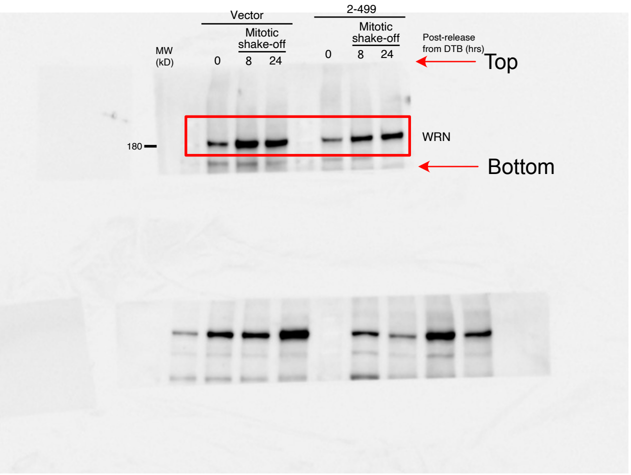

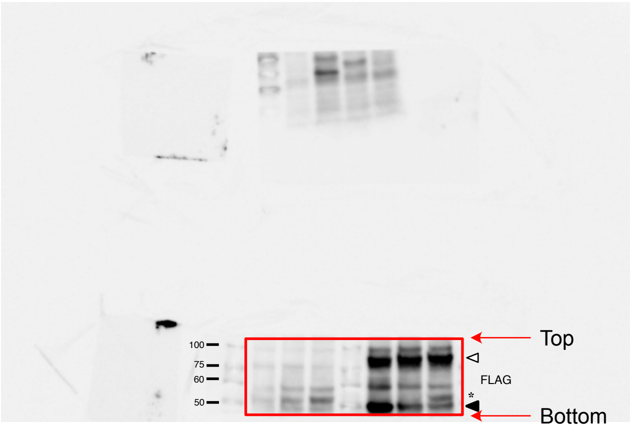

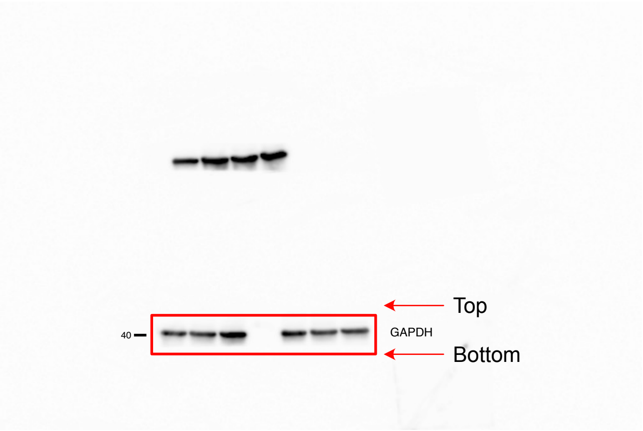

Supplementary Information for Fig. S3

Figure S3d

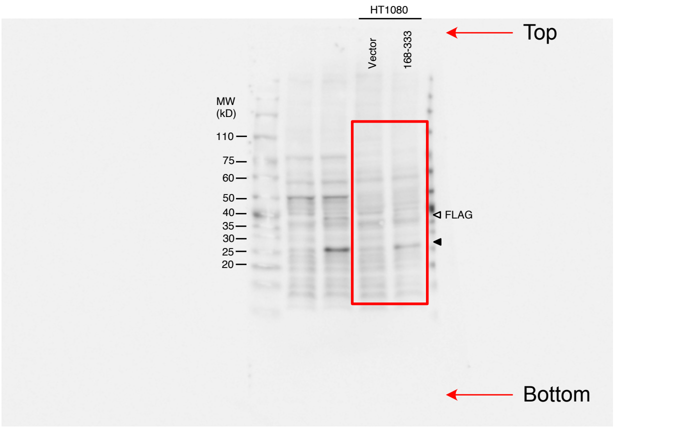

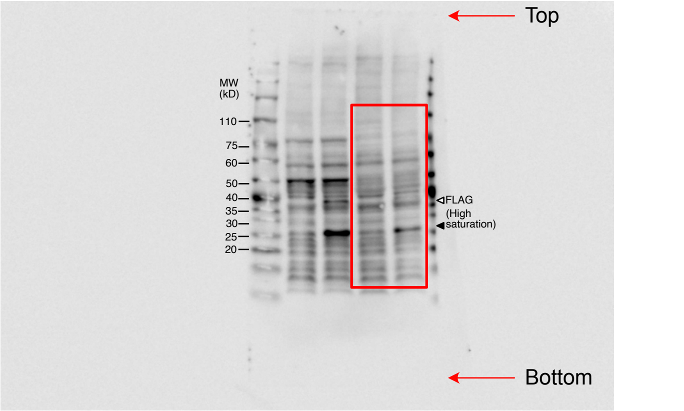

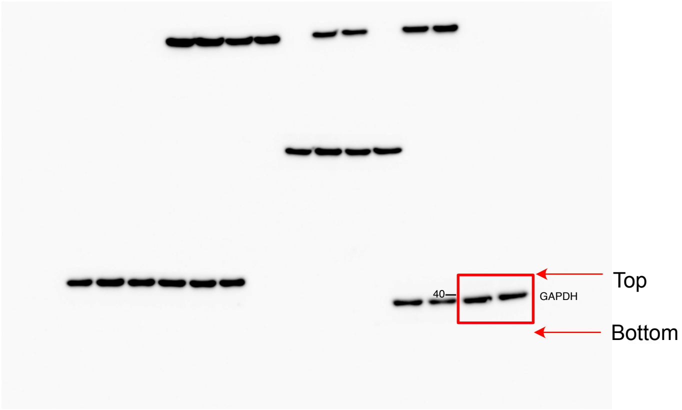

Supplementary File, related to AlphaFold2 analysis

Program config:


```
{
    "num_queries": 1,
    "use_templates": false,
    "use_amber": false,
    "msa_mode": "MMseqs2 (UniRef+Environmental)",
    "model_type": "AlphaFold2-ptm",
    "num_models": 5,
    "num_recycles": 3,
    "num_ensemble": 1,
    "model_order": [
        1,
        2,
        3,
        4,
        5
    ],
    "keep_existing_results": false,
    "rank_by": "plddt",
    "pair_mode": "unpaired+paired",
    "host_url": "https://api.colabfold.com",
    "stop_at_score": 100.0,
    "stop_at_score_below": 0,
    "recompile_padding": 1.0,
    "recompile_all_models": false,
    "commit": "9d4b35139f2a062b2eb8f8f9624eaaa703541dda",
    "is_training": false,
    "version": "1.3.0"
}
```


Input sequence

KLKCTETWSLNSLVKHLLGKQLLKDKSIRCSNWSKFPLTEDQKLYAATDAYAGFIIYRNLE
ILDDTVQRFAINKEEEILLSDMNKQLTSISEEVMDLAKHLPHAFSKLENPRRVSILLKDISE
NLYSLRRMIIGSTNIETELRPSNNLNLLSFEDSTTGGVQQKQI

coverage

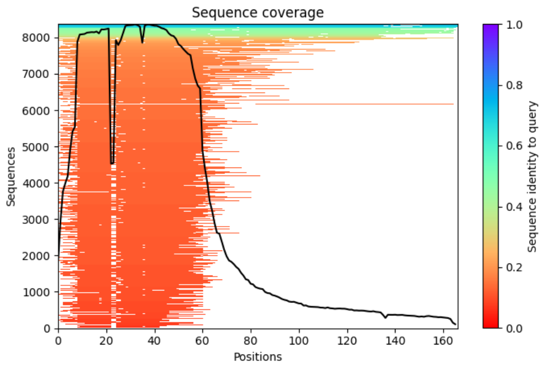

plddt

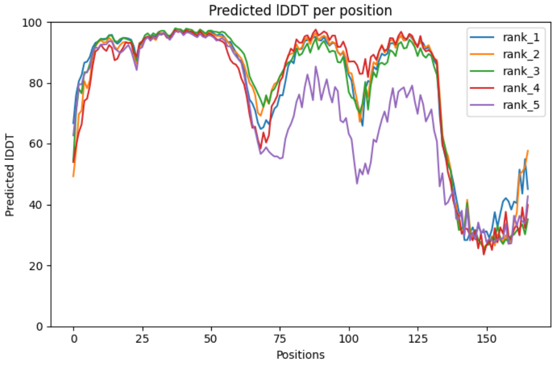

PAE

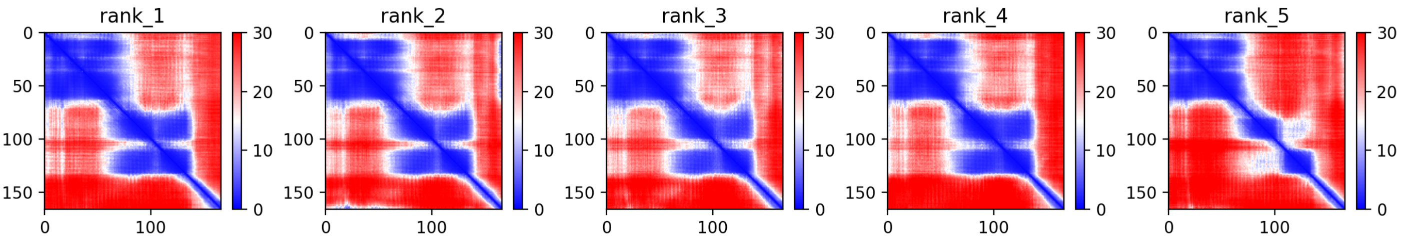

Supplement: Supplementary file 1 — Supplementary Information. [file 41598_2023_27598_MOESM1_ESM.pdf]
